# SMAD6 overexpression leads to accelerated myogenic differentiation of *LMNA* mutated cells

**DOI:** 10.1038/s41598-018-23918-x

**Published:** 2018-04-04

**Authors:** Alexandre Janin, Delphine Bauer, Francesca Ratti, Camille Valla, Anne Bertrand, Emilie Christin, Emilie Chopin, Nathalie Streichenberger, Gisèle Bonne, Vincent Gache, Tatiana Cohen, Alexandre Méjat

**Affiliations:** 1 University of Lyon, University of Lyon1 Claude Bernard Lyon1, Institut NeuroMyoGene, CNRS UMR5310, INSERM U1217 Lyon, France; 2 Laboratoire de Cardiogénétique Moléculaire, Centre de Biologie et Pathologie Est, Hospices Civils de Lyon, F-69677 Bron, France; 3 Sorbonne Université, INSERM UMRS_974, Center of Research in Myology, 75013 Paris, France; 4 Centre de Biotechnologie Cellulaire, Centre de Biologie et Pathologie Est, Hospices Civils de Lyon, F-69677 Bron, France; 5 Centre de Neuropathologie Est, Centre de Biologie et Pathologie Est, Hospices Civils de Lyon, F-69677 Bron, France; 6 Research Center for Genetic Medicine, Children’s National Medical Center, 111 Michigan Avenue NW, Washington, DC 20010 USA

## Abstract

*LMNA* gene encodes lamins A and C, two major components of the nuclear lamina, a network of intermediate filaments underlying the inner nuclear membrane. Most of *LMNA* mutations are associated with cardiac and/or skeletal muscles defects. Muscle laminopathies include Emery-Dreifuss Muscular Dystrophy, Limb-Girdle Muscular Dystrophy 1B, *LMNA*-related Congenital Muscular Dystrophy and Dilated Cardiomyopathy with conduction defects. To identify potential alterations in signaling pathways regulating muscle differentiation in *LMNA*-mutated myoblasts, we used a previously described model of conditionally immortalized murine myoblasts: H-2K cell lines. Comparing gene expression profiles in wild-type and *Lmna*^∆8–11^ H-2K myoblasts, we identified two major alterations in the BMP (Bone Morphogenetic Protein) pathway: Bmp4 downregulation and Smad6 overexpression. We demonstrated that these impairments lead to *Lmna*^∆8–11^ myoblasts premature differentiation and can be rescued by downregulating Smad6 expression. Finally, we showed that BMP4 pathway defects are also present in myoblasts from human patients carrying different heterozygous *LMNA* mutations.

## Introduction

*LMNA* gene encodes lamin A and C, two major components of the nuclear lamina, a network of intermediate filaments covering the inner nuclear membrane. These proteins can assemble into polymers beneath the nuclear envelope where they are crucial for the maintenance of interphase nuclear architecture, integrity of the nuclear envelope and chromatin structure^1–4^. They also interact with chromatin, transcription factors and epigenetic modifiers, thus playing an important role in the regulation of gene expression related to cell proliferation and differentiation, DNA replication and repair, and chromatin organization^5^.

Mutations in *LMNA* gene are associated with at least a dozen of inherited diseases, collectively called laminopathies^6^. Despite *LMNA* ubiquitous expression, most laminopathies involve tissue-specific phenotypes. *LMNA* mutations could lead to accelerated aging syndrome (Hutchinson-Gilford progeria syndrome), to abnormalities in adipose tissue (Dunnigan-type familial partial lipodystrophy), or in peripheral nerve (Charcot-Marie-Tooth type 2). Nevertheless, most of *LMNA* mutations are associated with cardiac and/or skeletal muscles defects^7^. Striated muscle laminopathies include Emery-Dreifuss Muscular Dystrophy (EDMD), Limb-Girdle Muscular Dystrophy 1B, *LMNA*-related Congenital Muscular Dystrophy (L-CMD) and Dilated Cardiomyopathy with conduction defects (DCM-CD). These four diseases are considered as a spectrum of the same pathology because of overlapping clinical features^8^.

Several mouse models have been generated to investigate the potential roles of lamins in skeletal muscle^9–15^. Based on findings using these models, several hypotheses have been proposed to explain the muscle phenotype in laminopathies: higher susceptibility to mechanical damages^16,17^, defective recruitment of nuclei at the neuromuscular junction (NMJ)^9^, alterations of gene expression and signaling pathways^3,17–19^ and impairment of muscle differentiation^20^. Previous reports have shown that *Lmna*^∆8–11^ mouse, previously thought to be a *Lmna*-null mouse^21^, represents a pertinent model to identify potential human pathophysiological mechanisms^15^. More particularly, based on *Lmna*^∆8–11^ (hereafter referred to as *Lmna*^−/−^) primary myoblasts, it has been suggested that A-type lamins deficiency caused an impairment in the muscle differentiation kinetics and that this deficiency is, in part, due to decreased endogenous level of other critical myoblast proteins^20^.

To avoid issues associated with primary myoblasts accessibility and to tightly control the kinetics of differentiation, we used a previously described model of conditionally immortalized murine myoblasts: wild-type and *Lmna*^−/−^ H-2K cell lines^18,22,23^.

Comparing gene expression profiles in wild-type and *Lmna*^−/−^ H-2K myoblasts, we identified several alterations of the BMP pathway. BMP (Bone Morphogenetic Protein) proteins were initially found to be crucial for osteogenesis^24,25^. They were further shown to be implicated in several other biological processes such as hematopoiesis, neuronal development or iron metabolism^26^. Finally, BMP is also a pivotal pathway regulating embryonic and foetal myogenesis. *In vitro*, this pathway promotes myoblasts proliferation and avoids premature differentiation^27^. *In vivo*, BMP signaling has been shown to be implicated in muscle growth, maintenance, and the balance between hypertrophy and atrophy by negatively regulating atrogenes such as *Fbx*2*0*^28^.

Our results identify two major alterations in the BMP pathway in *Lmna*^−/−^ myoblasts: Bmp4 downregulation and Smad6 overexpression. We demonstrate that these impairments lead to *Lmna*^−/−^ myoblasts premature differentiation and can be rescued by downregulating Smad6 expression. Finally, we showed that BMP4 pathway defects are also present in myoblasts from patients carrying different heterozygous *LMNA* mutations.

## Material and Methods

### Patient biopsies and culture of human myoblasts

Patient muscle samples were obtained from surgical biopsies of the deltoid muscle primarily performed for diagnosis purpose in accordance with principles of Helsinki declaration. All patients signed an informed consent for research. Human myoblasts were derived from four unrelated patients presenting different striated muscle laminopathies (Supplementary Table S1) with the following *LMNA* mutations: *LMNA* p.Lys32del (from 2 different patients, hereafter referred to as ΔK32 and *LMNA* p.Leu380Ser (hereafter referred to as L380S). Those myoblasts were collected and prepared as previously described^17^. Muscle samples and associated data from patient (*LMNA* Q310*: Z717 M01 and *LMNA* H222P: M45876) and healthy control (Y327 M01) were obtained from Cardiobiotec Biobank (CRB-HCL, Hospices Civils de Lyon, reference BB-0033–00046), a center for biological resources authorized by the French Ministry of Social Affairs and Health. All samples were collected and used in accordance with the ethical rules of the Biobank and in agreement with French legislation. The protocol was approved by local ethics committee and French Biomedicine Agency (reference DC2015–25–66). All experiments were performed in accordance with relevant guidelines and regulations. Myoblasts with *LMNA* Q310* and H222P mutations were purified by the Cellular Biotechnology Center (Centre de Biologie et Pathologie Est, Hospices Civils de Lyon). The purity of the population was evaluated by the proportion of desmin-positive cells in an immunofluorescence assay (Supplementary Figure S2). Cells were maintained in a HAM-F10 medium supplemented with 20% of FBS and 1% penicillin/streptomycin at 37 °C with 5% CO2.

### H-2K Cell culture

H-2K myoblasts^23^ were maintained at +33 °C and 10% CO_2_ in a proliferation medium composed of DMEM High glucose medium (HyClone) supplemented with 20% (v/v) fetal bovine serum (GE Healthcare), 2% (v/v) chick embryo extract (Eurogentec), 2% (v/v) glutamin (HyClone) and 1% (v/v) penicillin/streptomycin (growth medium) and complemented with 0.2% interferon-gamma (Sigma-Aldrich). H-2K were differentiated at +37 °C and 5% CO_2_ in DMEM High glucose medium supplemented with 2% (v/v) Horse Serum (HyClone) and 1% (v/v) penicillin/streptomycin. Proliferating H-2K myoblasts were grown on 100mm dishes coated with 0.01% gelatin. Differentiating cells were grown on matrigel-coated Nunc™ LAB-TEK® chamber slides (ThermoScientific) for immunofluorescence assays or matrigel-coated plastic dishes for RNA or protein assays.

### siRNA-mediated gene knockdown

H-2K cell lines were transfected either with *Smad6* siRNA or with *Lmna* siRNA and negative control siRNA (GeneSolution, Qiagen) at a final concentration of 10nM using lipofectamine RNAiMAX (Invitrogen), according to the manufacturer’s instructions.

### RNA isolation, microarrays and real-time quantitative PCR

Total RNA was isolated from H-2K and human myoblasts by column extraction (NucleoSpin RNA kit, Macherey Nagel) according to manufacturer’s instructions. Microarray analysis was performed by ProfileXpert facility (www.profilexpert.fr/). For each cell line, a triplicate of total RNA extract was analyzed. Microarray analysis was performed according to manufacturer’s instructions, on a MouseWG-6 V2.0 chip using an Illumina™ platform. The differentially expressed genes in wild-type and mutated groups were selected by unpaired t-test with significance level of *p* ≤ 0.05. Then, the genes with fold change less than 0.5 downregulated or greater than 2.0 upregulated between any two groups were selected for analysis.

For quantitative PCR, a maximum of 2 µg of total RNA was used for cDNA synthesis by reverse transcription PCR with random primers using the GoScript™ Reverse Transcription system (Promega) according to the manufacturer’s instructions. Real Time PCR was carried out with the FastStart Universal SYBR Green Master (Roche) kit using a Rotor-Gene from Corbett Life Science according to manufacturer’s instructions. Reactions were run in duplicate and *Hprt1* was used as housekeeping gene to control variability in expression level. *Ppib* (encoding cyclophilin B) for mouse samples and *GAPDH* and for human samples were also tested as housekeeping gene and the results were comparable. The primer sequences are provided in Supplementary data Table S3. To determine relative expression level, a standard curve based on dilutions of an internal sample of the assay was performed for each run and each gene.

### Western Blotting

Cells were maintained in proliferation or differentiation media, washed with PBS and harvested by trypsination and pellet by centrifugation at 1500 rpm during 5 min at +4 °C. After one more wash step, a dry pellet was obtained and stored at −80 °C. To extract proteins, the pellet was dissolved in extraction buffer based on classic RIPA buffer (25 mM Tris-HCL, 50 mM NaCl, 0.5% deoxycholic acid, 2% Nonidet P-40, 0.2% SDS) supplemented with a protease inhibitors cocktail (cOmplete from Roche) and phosphatases inhibitors (PhosSTOP from Roche). The lysis was performed on ice during 30 minutes. After sonication, protein concentration was determined by a Lowry-like assay using the DC protein Assay from BioRad. Dosages were performed according to manufacturer’s specifications. An amount of 20 µg of total lysate was loaded onto 12% precasted MiniPROTEAN® TGX gels (Bio-Rad). Transfer was performed onto 0.22 µm PVDF membranes. After being blocked in 5% non-fat dried milk dissolved in TBS 1X, membranes were incubated overnight with primary antibodies (see Supplementary Table S4). After three wash steps with TBS, membranes were probed one hour with appropriate secondary antibodies. Following three new wash steps with TBS, membranes were developed using the ECL reagent (GE Healthcare) or ECL2 reagent (ThermoScientific) on Fuji Medical X-Ray Films (FujiFilm).

### Immunostaining

Cells were fixed for 10 minutes in 4% paraformaldehyde diluted in PBS. After three wash steps with PBS, cells were permeabilized for 10 min in PBS 1X with Triton™-X100 0.1% and then blocked in PBS-BSA 1% for 10 minutes. Cells were stained with primary antibodies, diluted in blocking solution, during 2 hours at room temperature. Following three wash steps in PBS, cells were probed with appropriate secondary antibodies, coupled to AlexaFluor probes, diluted in blocking solution for 1 hour at room temperature. Immunofluorescence images were acquired with a Zeiss AxioImagerZ1 microscope equipped with two coolsnap cameras. Images were acquired using Metamorph software and analyzed using ImageJ software.

### Statistical analysis

Non parametric Mann Whitney test was performed using GraphPad Prism version 6.00 for Mac (GraphPad Software, La Jolla California USA, www.graphpad.com) (*p < 0.05, **p < 0.01, ***p < 0.001, ****p < 0.0001).

### Data availability

The datasets generated during and/or analyzed during the current study are available from the corresponding author on reasonable request.

## Results

### Transcriptomic analysis identifies Bmp pathway alterations in Lmna^−/−^ myoblasts

H-2K immortalized myoblasts (MB), isolated from Immortomice^TM^ ^22,23^, have been previously shown to be a powerful model combining the ability to obtain a large number of myoblasts and a tight control of their differentiation^29^. H-2K myoblasts express a thermosensitive form of SV-40 large T antigen under the control of an IFNγ-responsive promoter. They are kept in proliferation at 33 °C in presence of IFNγ or switched to differentiation into myotubes at 37 °C in absence of IFNγ. *Lmna*^−/−^ H-2K myoblasts and their wild-type (WT) littermate controls have been previously obtained by crossing *Lmna*^−/−^ mice and Immortomice^TM^ ^18^.

**Figure 1:**
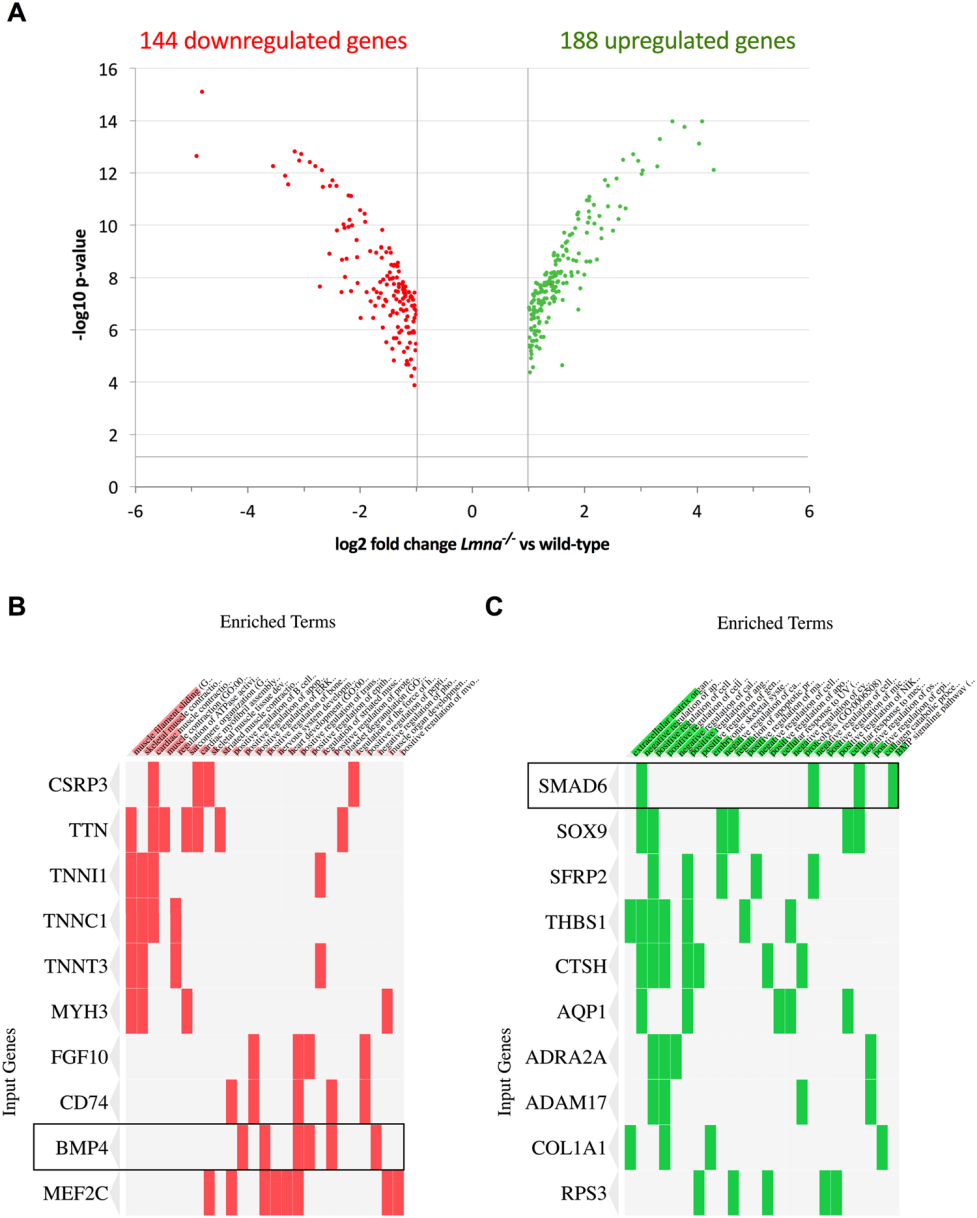
Transcriptomic analysis of *Lmna*^−/−^ *versus* wild-type H-2K myoblasts expression profiles. (**A**) Volcano plot displaying the repartition of up- and down-regulated genes in *Lmna*^−/−^ H-2K myoblasts compared to wild-type. Only the genes with a p-value inferior to 0.05 (−log10 p-value superior to 1.3) and at least a twofold change (log2 fold-change superior to 1) are displayed. (**B**,**C**) Clustergramm listing downregulated (**B**) and upregulated (**C**) genes were generated using ENRICHR (http://amp.pharm.mssm.edu/Enrichr/) to identify misregulated genes of interest in enriched GO Biological Process 2017 categories.

In order to seek alterations in gene expression induced by *Lmna* inactivation, we performed a transcriptomic analysis on *Lmna*^−/−^ and wild-type H-2K myoblasts (MB). We found 144 downregulated genes and 188 upregulated genes in *Lmna*^−/−^ compared to wild-type MB (p-value < 0.05) displaying at least a two-fold change in their relative expression (Fig. 1A and Supplemental Table S5). ENRICHR^30,31^ was used to determine the biological processes associated to these gene sets. Many down-regulated genes are involved in muscle contraction and sarcomere organization as well as in skeletal muscle differentiation (Fig. 1B and Supplemental Table S5). On the other hand, upregulated genes are related to extracellular matrix organization, apoptosis, cell migration or proliferation (Fig. 1C and Supplemental Table S6). Clustergramms based on these gene sets allowed us to identify two impairments that belong to the same pathway: *Bmp4* gene in the downregulated group and *Smad6* in the upregulated group (Fig. 1B and C).

Taken together, these results suggest that Bmp pathway, and more particularly *Bmp4* and *Smad6* gene expression, could be altered in *Lmna*^−/−^ myoblasts.

### Loss of Lamin A/C alters Bmp4 secretion but not Bmp receptors expression

**Figure 2:**
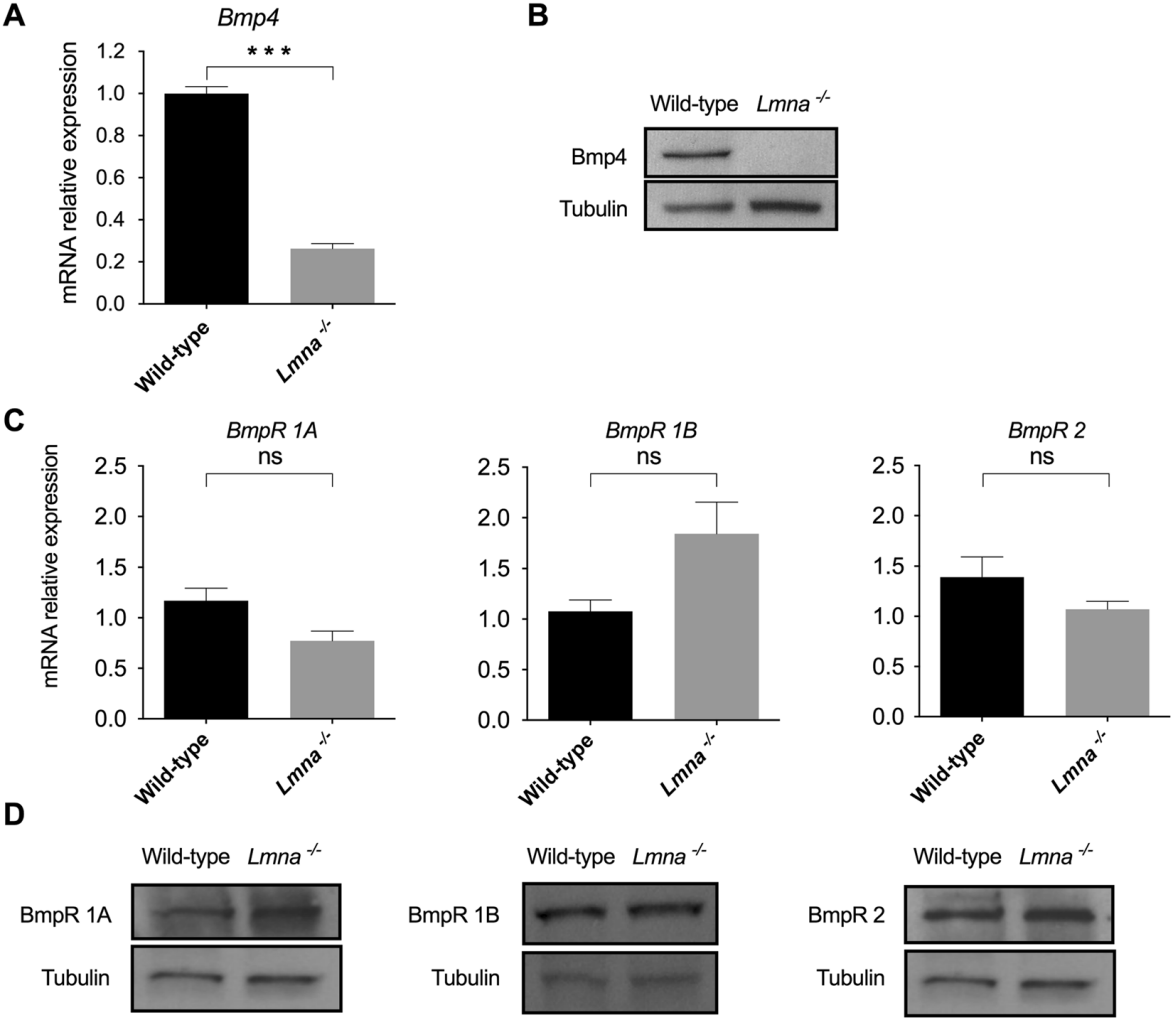
Bmp4 and Bmp receptors (BmpR) expression in *Lmna*^−/−^ vs wild-type H-2K myoblasts. (**A**,**C**) Expression of *Bmp4* (**A**) and Bmp receptors (*BmpR1A*, *1B* and *2*) (**C**) measured by quantitative reverse transcription polymerase chain reaction (qRT-PCR) in wild-type and *Lmna*^−/−^ H-2K MB. Expression is shown as mean (±SEM) of at least three biological replicates and two technical replicates. Values are relative expression normalized to *Hprt1* housekeeping gene expression. (**B**,**D**) Protein levels of Bmp4 (**B**) and Bmp receptors (**D**) quantified by western blot in *Lmna*^−/−^ and wild-type H-2K MB. Protein levels of tubulin in each sample are provided as normalizer. (*p < 0.05, **p < 0.01, ***p < 0.001). Full-length blots are presented in Supplementary Figure 2.

According to the transcriptomic data, *Bmp4* expression is decreased by 81% in *Lmna*^−/−^ myoblasts compared to wild-type (Supplementary dataset). To validate this result, we measured *Bmp4* expression by quantitative PCR and western blot. We observed a 78% decrease of *Bmp4* mRNA level relative to *Hprt1* expression in *Lmna*^−/−^ MB (Fig. 2A). This decrease was further confirmed with a second pair of primers (data not shown). Unexpectedly, we could not detect any band corresponding to Bmp4 protein by western blot in proliferating *Lmna*^−/−^ myoblasts (Fig. 2B), suggesting that no Bmp4 is produced by them (Fig. 2B). Moreover, the decrease of *Bmp4* mRNA level relative to *Hprt1* expression was also observed following *Lmna* knock-down using siRNA in H-2K wild-type MB (Supplementary Figure S7). Interestingly, at the opposite, *Lmna* overexpression in H-2K *Lmna*^−/−^ MB is able to restore *Bmp4* expression at levels found in wild-type H-2K MB (Supplementary Figure S8).

Bmp4 binds to type 1 and type 2 transmembrane receptors (respectively BmpR1A, 1B and 2) and activate the downstream cascade^24^. In order to further explore the pathway, quantitative PCR and western blots were performed to quantify the expression of *Bmp* receptors. Both at the transcriptional and at the protein levels, BmpR1A, BmpR1B and BmpR2 expression levels in *Lmna* null myoblasts are normally expressed in both wild-type and *Lmna*^−/−^ cells (Fig. 2C and D) suggesting that these effectors are not affected by *Lmna* loss.

### Smad6 expression is upregulated in *Lmna*^−/−^ myoblasts

The formation of BmpR1 and 2 complexes at the membrane allows the constitutive kinase activity of BmpR2 to phosphorylate BmpR1. After phosphorylation-mediated activation, BmpR1 phosphorylates the Receptor-regulated Smads (R-Smads): Smad1, Smad5 and Smad8 (pSmad1/5/8)^32^. Phosphorylated R-Smads form a heteromeric complex either with the Common mediator Smad (Co-Smad) Smad4 able to translocate into the nucleus in order to activate Smad-target genes or with Smad6, an Inhibitory Smad (I-Smad), that is retained in the cytoplasm^26,33,34^.

**Figure 3:**
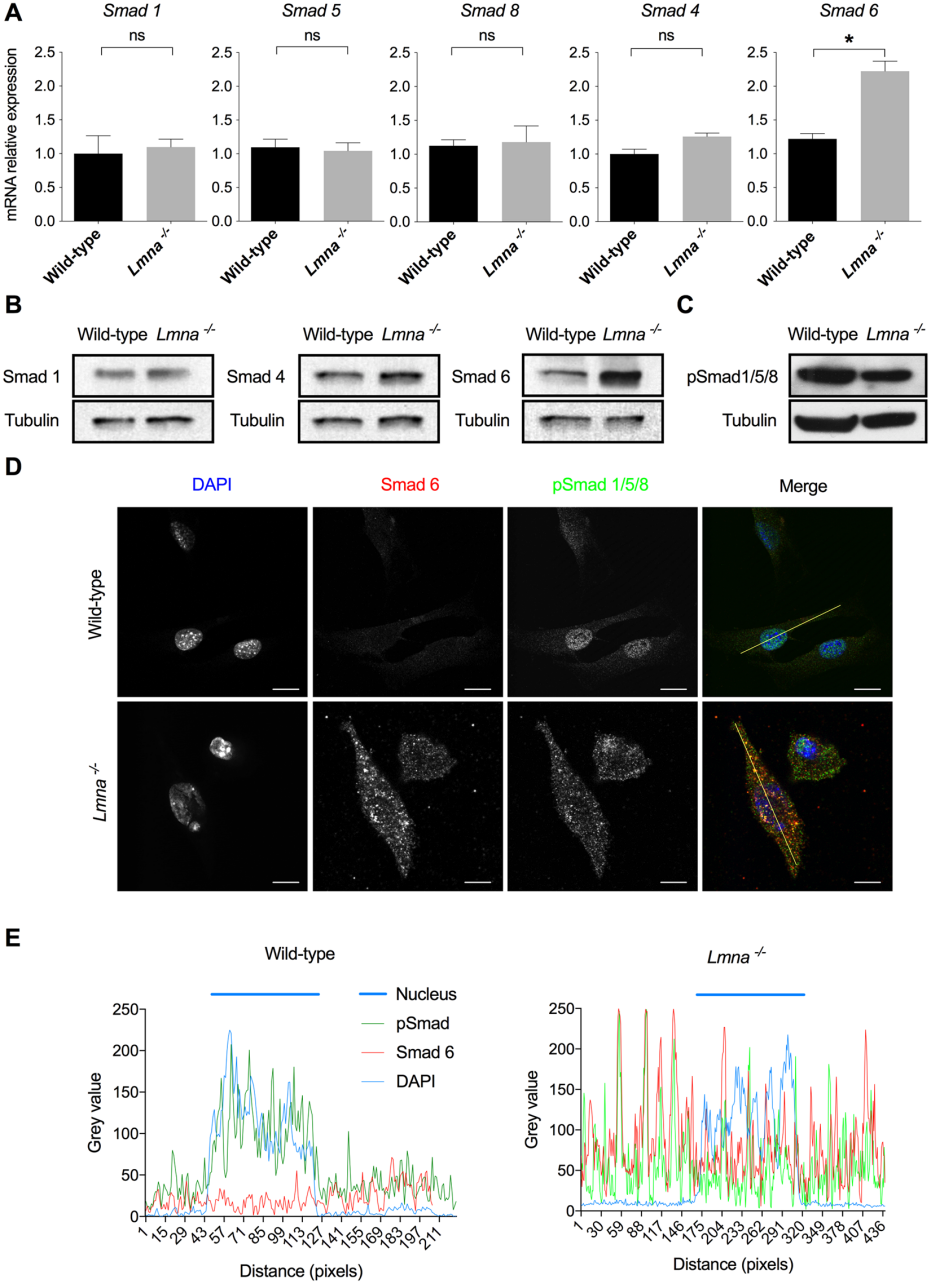
Smads expression and intracellular localization. (**A**) Expression of *Smad1*, 5, 8, 4 and 6 measured by quantitative reverse transcription polymerase chain reaction (qRT-PCR) in wild-type and *Lmna*^−/−^ H-2K MB, at the proliferative stage. Expression is shown as mean (±SEM) of at least three biological replicates and two technical replicates. Values are relative expression normalized on *Hprt1* housekeeping gene expression. (**B**) Protein levels of Smad1, Smad4 and Smad6 quantified by western blot in *Lmna*^−/−^ H-2K MB versus wild-type, at the proliferative stage. (**C**) Phosphorylation levels of total Smad1/5/8 measured by western blot. (**B**,**C**) Protein levels of tubulin in each sample are provided as normalizer. (**D**) Immunofluorescence assay in wild type and *Lmna*^−/−^ H-2K MB, at the proliferative stage, showing the intracellular localization of phosphorylated R-Smad and Smad6. Scale bar: 10 µm. (**E**) Intensity plots of each dye along the yellow line shown in (**D**). (*p < 0.05, **p < 0.01, ***p < 0.001). Full-length blots are presented in Supplementary Figure 3.

To further explore potential defects in the Bmp4 pathway, expression of R-Smads (i.e. Smad1, 5 and 8), Co-Smad Smad4 and I-Smad Smad6 were quantified by quantitative PCR and western-blot (when antibodies were available). No defect in R-Smads and Smad4 expression, neither at the transcription level nor at the protein level, could be observed (Fig. 3A and B). However, in line with the transcriptomic data (Fig. 1C), a two-fold increase in *Smad6* expression was measured in *Lmna*^−/−^ compared to wild-type myoblasts. The relative mRNA level is 1.22 ± 0.08 in WT H-2K *versus* 2.22 ± 0.15 in *Lmna*^−/−^ H-2K MB (mean ± SEM) (Fig. 3A). This twofold mRNA level increase translated into an accumulation of Smad6 protein was confirmed by western blot (Fig. 3B).

Moreover, the increase of *Smad6* mRNA level was also observed following *Lmna* knock-down using siRNA in H-2K wild-type MB (Supplementary Figure S7). Interestingly, at the opposite, *Lmna* overexpression in H-2K *Lmna*^−/−^ MB is able to normalize *Smad6* expression compared to wild-type H-2K MB (Supplementary Figure S8).

### Overexpressed Smad6 sequesters pSmad1/5/8 in the cytoplasm

The phosphorylation level of R-Smads (phosphorylated Smad1/5/8) was determined by western blot. Interestingly, despite the absence of detectable Bmp4 ligand, the level of phosphorylation of R-Smads is only slightly decreased in H-2K *Lmna*^−/−^ cells (Fig. 3C). This result suggests a possible compensation by other pathways using the phosphorylation of Smads 1, 5 and 8 as cellular transducers.

The inhibitor Smad6 has been shown to compete with Smad4 to interact with the pSmad1/5/8 complex^34^ resulting in a Smad6-pSmad1/5/8 complex unable to translocate into the nucleus to activate Smad target genes transcription. For this reason, the intracellular localization of Smad6 and phosphorylated R-Smads were evaluated by immunofluorescence (Fig. 3D). In wild-type myoblasts, pSmad1/5/8 were almost exclusively observed in the nucleus of H-2K cells whereas only a faint signal corresponding to Smad6 could be detected in the cytoplasm. In contrast, pSmad1/5/8 were mainly detected in the cytoplasm of *Lmna*^−/−^ myoblasts where they tend to colocalize with Smad6 dots (Fig. 3D). To better characterize these changes in Smad localizations, the intensity (grey value) of each labelling was quantified along a line following the cell main axis (Fig. 3E). This confirmed that in wild-type cells, the labelling (in green) of phosphorylated R-Smads colocalized with the labeling of cell’s nuclei (DAPI staining in blue). In *Lmna*^−/−^ myoblasts, phosphorylated R-Smad signals are dispersed throughout the cell and pSmad1/5/8 and Smad6 intensity peaks colocalize (Fig. 3E). These results suggest that Smad6 overexpression leads to a sequestration of Smad6-pSmad1/5/8 complex in the cytoplasm of *Lmna*^−/−^ myoblasts (Fig. 3D and E).

### Altered Bmp4 pathway leads to accelerated muscle differentiation

After translocation into the nucleus, the Smad4-pSmad1/5/8 complex is able to induce the transcription of target genes such as *Id1* and *Id2* (for Inhibitor of differentiation/DNA Binding)^35,36^. These proteins bind to ubiquitous E-proteins to form inactive heterodimers. When associated with Id proteins, E-proteins cannot bind and activate muscle-specific transcription factors such as MyoD1, blocking muscle differentiation^37^. Consequently, Id proteins expression prevents premature activation of differentiation factors. In skeletal muscle, Bmp pathway has been shown to promote muscle progenitors expansion and prevent premature muscle differentiation^27^.

To evaluate if Bmp4 downregulation and Smad6 overexpression could affect *Lmna*^−/−^ myoblasts differentiation, *Lmna*^−/−^ *vs* wild-type H-2K cells were fixed and immunolabelled for Myosin Heavy Chain (MyHC, MF-20), a marker of differentiation, forty-eight hours after switching in differentiation conditions.

**Figure 4:**
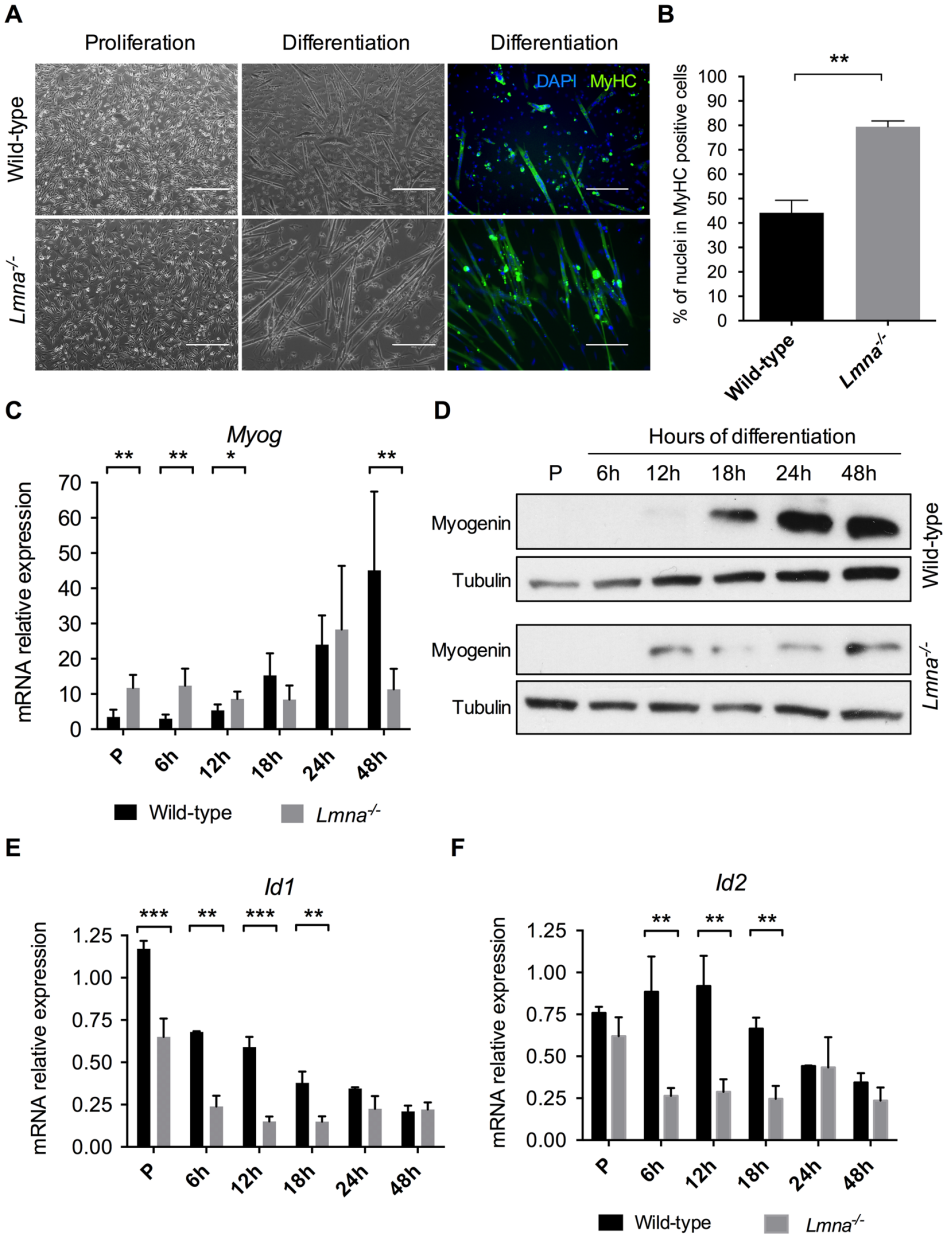
*Lmna*^−/−^ myoblasts premature differentiation. (**A**) Phase-contrast photographs and immunofluorescence labelling of MyHC (in green) and DAPI (in blue) of wild-type and *Lmna*^−/−^ H-2K MB in proliferation and after 48 hours of differentiation. Scale bar: 400 µm for proliferation and 200 µm for differentiation. (**B**) Quantification of the differentiation index (percentage of nuclei in MyHC positive cells) in wild-type and *Lmna*^−/−^ H-2K myotubes after 48 hours of differentiation. (**C**,**D**) Expression of Myogenin measured by quantitative reverse transcription polymerase chain reaction (qRT-PCR) (**C**) and western blot (**D**) in wild-type and *Lmna*^−/−^ proliferating myoblasts (P, Proliferation) and during muscle differentiation (from 6 hours to 48 hours of differentiation). (**E**,**F**) Expression of *Id1* (**E**) and *Id2* (**F**) measured by qRT-PCR in wild-type and *Lmna*^−/−^ proliferating myoblasts (P, Proliferation) and during muscle differentiation (from 6 hours to 48 hours of differentiation). Expression is shown as mean (± SEM) of biological replicates (n = 4). Values are relative expression normalized on *Hprt1* housekeeping gene expression. (*p < 0.05, **p < 0.01, ***p < 0.001, ****p < 0.0001). Full-length blots are presented in Supplementary Figure 4.

Phase-contrast photographs and immunofluorescences (Fig. 4A) revealed accelerated differentiation in H-2K *Lmna*^−/−^ myotubes compared to wild-type. H-2K *Lmna*^−/−^ myotubes (MT) appeared longer but thinner than wild-type. The differentiation index (DI), i.e. the percentage of nuclei in MyHC positive myotubes, was calculated for both conditions and this index is significantly increased in H-2K *Lmna*^−/−^ MT: after forty-eight hours in differentiation medium, 79% (DI = 79 ± 2) of nuclei were included in a MT (MyHC positive cells) in the *Lmna*^−/−^ condition versus 44% in the control condition (DI = 44 ± 5) (Fig. 4B).

In order to better characterize this anticipated differentiation process, the expression of Myogenin, a crucial muscle-specific transcription factor, was measured at both the transcriptional and protein levels during differentiation. Whereas Myogenin mRNA only started to be expressed in wild-type myoblasts after 12 hours of differentiation, it could already be detected in proliferating and early differentiating (6 hours) *Lmna*^−/−^ H-2K cells (Fig. 4C). *Myog* expression reached a plateau after 24 hours in *Lmna*-null muscle cells while it still increased in wild-type cells after 48 hours. This precocious expression could also be observed at the protein level where Myogenin was already detectable after 12 hours of differentiation in mutated versus 18 hours in wild-type myoblasts (Fig. 4D). Interestingly, although *Myog* appeared earlier, its expression did not increase with differentiation and remained low compared to WT by 48 hours.

Altered *Myog* expression correlated with the decrease of *Id1* and *Id2* expression, target genes of the Bmp4 pathway, in *Lmna*^−/−^ H-2K cells compared to wild-types during differentiation (Fig. 4E and F). *Id1* expression was already reduced by 50% in proliferating *Lmna*^−/−^ myoblasts and about 70 to 80% during differentiation (Fig. 4E). *Id2* expression was not affected in proliferating myoblasts but immediately declined upon differentiation (Fig. 4F).

Taken together, these results demonstrate that *Lmna*^−/−^ H-2K myoblasts differentiate prematurely and that could be due to Bmp pathway alterations through *Id1* and *Id2* misregulation.

### Recombinant murine Bmp4 treatment maintains wild-type MB in proliferation but cannot prevent *Lmna*^−/−^ myoblasts differentiation

As Bmp receptors were normally expressed in *Lmna*^−/−^ myoblasts, we tested whether they could respond to Bmp4 addition.

**Figure 5:**
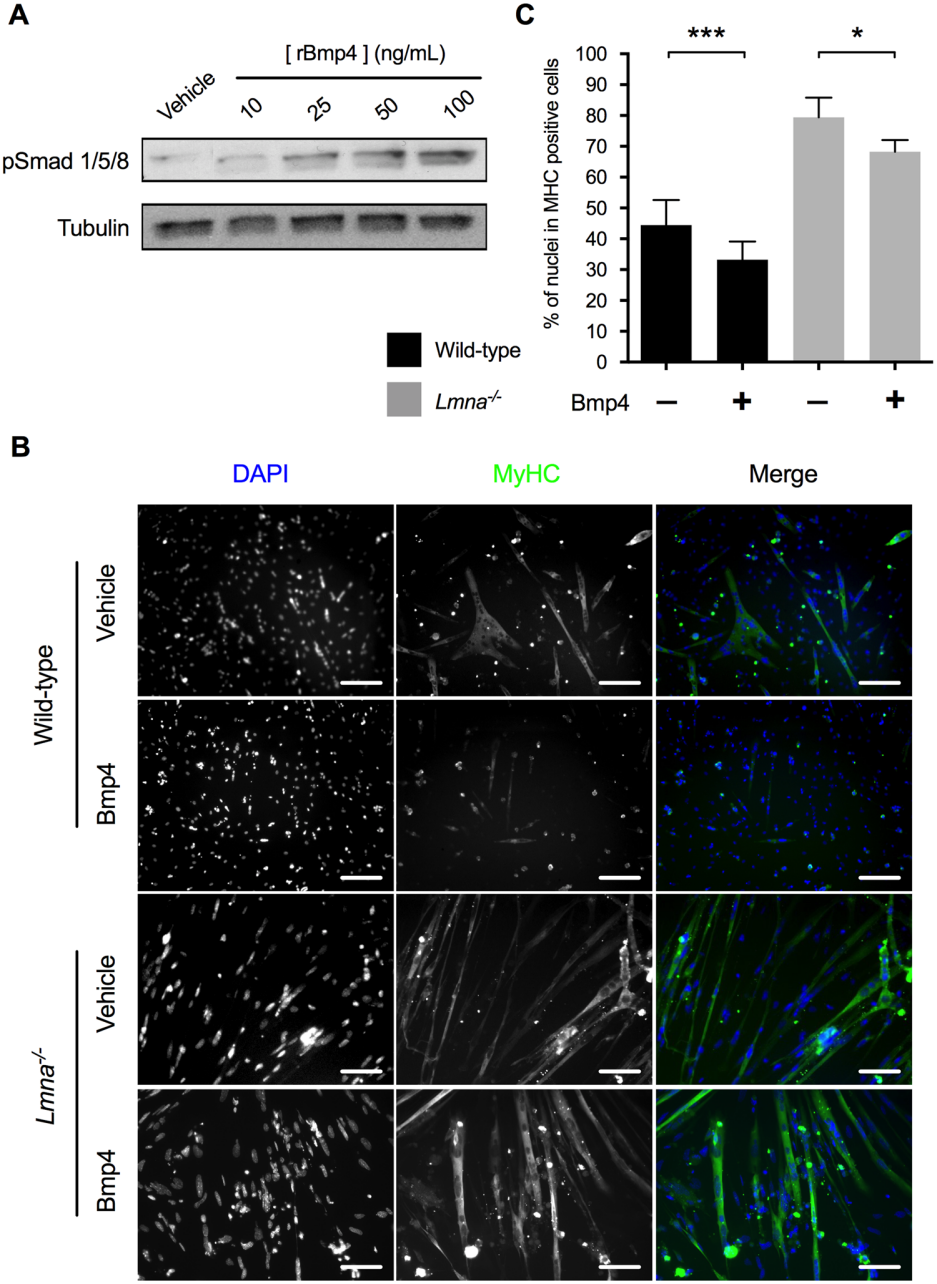
Wild-type and *Lmna*^−/−^ myoblasts response to recombinant Bmp4 addition. (**A**) Dose-response to recombinant Bmp4 treatment in wild-type H-2K MB measured by R-Smad phosphorylation. (**B**,**C**) Wild-type or *Lmna*^−/−^ myoblasts were first incubated during 24 hours in proliferation medium complemented with vehicle (Vh) or recombinant mouse Bmp4 (100 ng/mL) and then switched in differentiation conditions for 48 hours. (**B**) Immunofluorescence labelling of treated wild-type and *Lmna*^−/−^ H-2K MT after 48 hours of differentiation. Muscle nuclei were stained with DAPI (in blue) and differentiated myotubes were stained with MyHC antibody (in green). (**C**) Differentiation indexes (percentage of nuclei in MyHC positive cells) quantified in treated wild-type and *Lmna*^−/−^ H-2K. (*p < 0.05, **p < 0.01, ***p < 0.001, ****p < 0.0001). Full-length blots are presented in Supplementary Figure 5.

To determine the best concentration of recombinant Bmp4 to be used, a dose-response experiment was performed in wild-type H-2K MB (Fig. 5A). Increasing concentrations of recombinant Bmp4 induced an increase of R-Smads phosphorylation reaching a maximum level at the concentration of 100 ng/mL. Thus, this concentration has been used for all further experiments.

To evaluate if the addition of Bmp4 could rescue the premature muscle differentiation in *Lmna*^−/−^ H-2K MB, myoblasts were treated with recombinant Bmp4 (100 ng/mL) during 24 hours and then placed into differentiation medium during 48 hours without Bmp4. Cells were fixed, and immunolabelled for MyHC (in green) and DAPI (in blue) (Fig. 5B). Differentiation indexes were calculated for each condition (Fig. 5C). In wild-type myoblasts, the differentiation index decreased of 25% (from DI = 44 ± 5 in “vehicle condition” to DI = 33 ± 3 in “Bmp4-treated cells”), confirming that Bmp addition reduces myogenic differentiation. In mutated myoblasts, differentiation index only decreased of 14% (from DI = 79 ± 2 in the “vehicle condition” to DI = 68 ± 2 in the “Bmp4-treated cells”) and was still twice higher than in wild-type cells suggesting that *Lmna* null myoblasts cannot fully respond to Bmp4 and that the Bmp pathway may be blocked by a downstream effector.

### Smad6 inactivation is sufficient to prevent *Lmna*^−/−^ myoblasts premature differentiation and to restore their response to Bmp4

**Figure 6:**
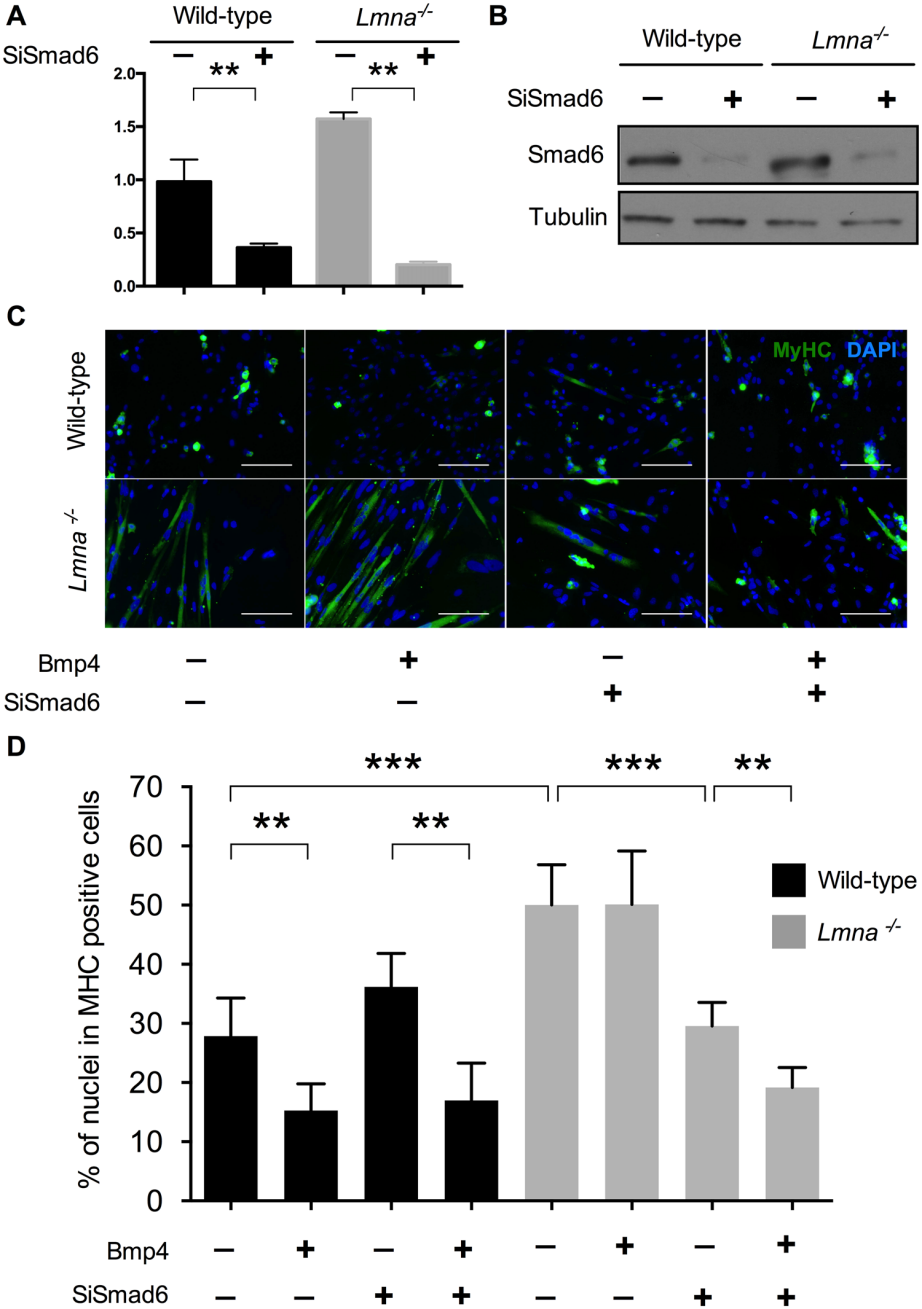
Precocious differentiation rescue in *Lmna*^−/−^ MB by *Smad6* inactivation. (**A**) Quantification of *Smad6* expression by RT-qPCR in presence of scramble siRNA (SiCtrl) or a SiRNA against Sma*d*6. Values are relative expression normalized on *Hprt1* housekeeping gene expression. (**B**) Smad6 protein levels quantified by western blot in presence of SiCtrl or SiSmad6. Tubulin expression levels are provided as normalizer. (**C**) Immunofluorescence assay showing MyHC (in green) and DAPI (in blue) of wild-type and *Lmna*^−/−^ H-2K myotubes after 24 hours of treatment with *Smad6*-SiRNA (or SiCtrl) and recombinant Bmp4 (100ng/mL) (or vehicle) followed by 48 hours of differentiation. Scale bar: 200 µm. (**D**) Differentiation index (percentage of nuclei in MyHC positive cells) for each condition showing that the double treatment by *Smad6*-SiRNA and recombinant Bmp4 in *Lmna*^−/−^ H-2K MB is able to rescue the phenotype and to prevent the observed premature differentiation. At least 350 nuclei per conditions were analyzed. (*p < 0.05, **p < 0.01, ***p < 0.001, ****p < 0.0001). Full-length blots are presented in Supplementary Figure 6.

Based on the previous observation on Bmp4, we tried to rescue *Lmna*^−/−^ premature differentiation by modulating the pathway with small interfering RNA against Smad6 (SiSmad6). *Smad6* expression decreased of about 75% in presence of SiSmad6, both at the RNA (Fig. 6A) and protein (Fig. 6B) levels, in wild-type and *Lmna*^−/−^ H-2K cells.

As previously, proliferating H-2K myoblasts were incubated during 24 hours with SiSmad6 (or a control siRNA) in combination with recombinant Bmp4 (or its vehicle) and then switched to differentiation without any treatment. After 48 hours of differentiation, cells were fixed and immunolabelled for MyHC (Fig. 6C) to calculate a differentiation index (DI) in each condition (Fig. 6D).

As before, Bmp4 addition significantly reduced (−45%) the differentiation index in wild-type myoblasts (from 28% ± 3 to 15% ± 2). At the opposite, Smad6 inactivation slightly increased the DI, although not significantly, (DI with SiSmad6: 36 ± 3). But the addition of Bmp4, even in presence of SiSmad6, lead to a reduced DI comparable to the one measured with Bmp4 without SiSmad6 (DI: 17 ± 3, −39% compared to SiSmad6 only). These results confirm that, in wild-type cells, Bmp4 acts as a repressor of muscle differentiation whereas Smad6 promotes it.

As expected and previously described in Fig. 5, Bmp4 addition (with control SiRNA) had not effect on *Lmna*^−/−^ H-2K differentiation. With or without Bmp4, the differentiation index was similar around 50% (±2 or ±3 respectively) and significantly higher than untreated (SiRNA control + vehicle) wild-type cells (DI: 28 ± 3), showing, once again, that *Lmna*-null myoblasts differentiate prematurely and do not respond to Bmp4. However, Smad6 down-regulation in *Lmna*^−/−^ myoblasts lead to a reduced differentiation index (−41%, DI: 30 ± 2) comparable to the untreated wild-type cells (Fig. 6D) and to the formation of large myotubes (Fig. 6C) suggesting that a normal differentiation is restored by Smad6 removal. Moreover, *Lmna*^−/−^ myoblasts treated with both SiSmad6 and Bmp4 showed a differentiation index of 19% (±1), significantly inferior to Smad6 knock-down only but comparable to wild-type Bmp4-treated cells demonstrating that Smad6 knock-down fully restored the Bmp pathway and response to Bmp4 addition in *Lmna*^−/−^ myoblasts.

These results demonstrate that Smad6 overexpression is responsible for *Lmna*^−/−^ premature differentiation and that timely differentiation can be restored by Smad6 knock-down.

### BMP4 pathway is also altered in human *LMNA*^+/−^ mutated myoblasts

**Figure 7:**
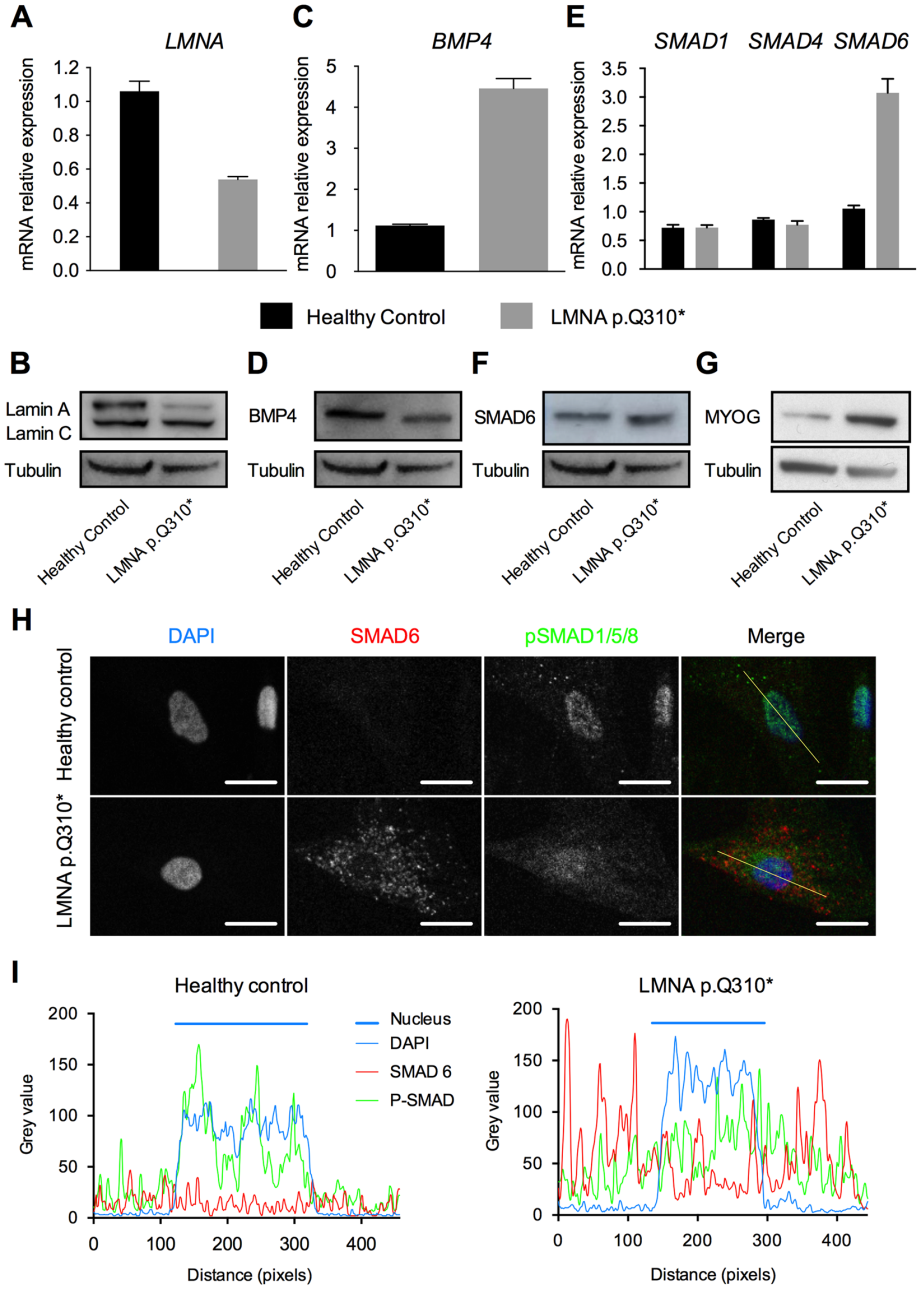
Impaired BMP4 pathway in Human myoblasts carrying a non-sense mutation (*LMNA* p.Q310*). (**A**,**C**,**E**) Expression of *LMNA*, *BMP4*, *SMAD1, SMAD4* and *SMAD6* measured by quantitative reverse transcription polymerase chain reaction (qRT-PCR) in MB from a healthy control and *LMNA* p.Q310* carrier. Expression is shown as mean of biological replicates (n = 2). Values are relative expression normalized on *HPRT* housekeeping gene expression. (**B**,**D**,**F**,**G**) Protein levels of Lamins A and C, BMP4, SMAD6 and Myogenin quantified by western blot in MB from control and patient. Tubulin expression levels are provided as normalizer. (**H**) Immunofluorescence assay in wild type and mutated MB showing the intracellular localization of phosphorylated R-SMADs and SMAD6. Scale bar: 10 µm. (**I**) Intensity plots of each dye along the line shown in (**C**). Full-length blots are presented in Supplementary Figure 7.

We sought to verify that BMP4 pathway alterations were also relevant for the human pathology. Hence, the main defects observed in murine *Lmna*^−/−^ myoblasts were probed in human myoblasts from patient carriers of different *LMNA* mutations. Three already reported mutations were tested: two missense mutations and an in-frame deletion reported in a typical EDMD family (p.H222P)^38^, and in patients with severe EDMD (p.K32del) or L-CMD (p.K32del and p.L380S)^39–41^. An additional mutation, not already reported in GnomAD consortium, was also tested. The patient is heterozygous for a nonsense mutation (NM_170707.2: c.928C>T), p.Q310*, located in exon 5 of *LMNA* gene. This 41-year old man suffered from cardiomyopathy associating sinus bradycardia, atrio-ventricular block and ventricular arrhythmia. This cardiac phenotype led to the implantation of a pacemaker. The patient suffered also from a myopathy associating widespread muscle pain, walking difficulties and chronic tiredness. Moving from a squat position to standing was difficult. Electromyogram argued in favor of muscular damages (Supplementary Table S1). The *LMNA* identified mutation leads to *LMNA* haploinsufficiency (Fig. 7A and B). *LMNA* mRNA expression was reduced of 49% (Fig. 7A) and correlated with a decrease of lamin A protein (Fig. 7B) in *LMNA*^+/Q310*^ myoblasts compared to myoblasts from an age- and gender-matched healthy control subject (45-year old man, ref Y327 M01).

BMP4 expression was quantified in the *LMNA* mutated-patients and healthy control myoblasts. Surprisingly, *BMP4* mRNA expression was four times higher in *LMNA*^+/Q310*^ MB compared to control myoblasts (1.12 ± 0.03 in control MB *versus* 4.6 ± 0.24 in *LMNA*^+/Q310*^ MB, mean +/− SEM) (Fig. 7C). However, at the protein level, a decrease of BMP4 expression was observed (Fig. 7D) suggesting that *LMNA* mutation also affects BMP4 protein stability. In other patient myoblasts, *BMP4* mRNA expression levels were variable but always altered (Supplementary Figure S9).

*SMAD6* mRNA was also found to be overexpressed in *LMNA*^+/Q310*^ myoblasts (1.06 ± 0.06 in control MB *versus* 3.07 +/−0.24 in *LMNA*^+/Q310*^ MB, mean ± SEM) although no difference in other SMAD (i.e. *SMAD1* and *SMAD4*) expression was found (Fig. 7E). SMAD6 overexpression in *LMNA*^+/Q310*^ myoblasts was confirmed at the protein level by western-blot (Fig. 7F). In other patient myoblasts, *SMAD6* mRNA expression was increased in *LMNA*^+/p.K32del^ and *LMNA*^+/p.L380S^ myoblasts but not in *LMNA*^+/H222P^ myoblasts (Supplementary Figure S9).

Finally, to determine if the molecular mechanism of the pathway impairments were comparable between H-2K and human myoblasts, an immunofluorescence to localize pSMAD1/5/8 and SMAD6 was performed in *LMNA*^+/Q310*^ myoblasts. As in *Lmna*^−/−^ H-2K myoblasts, the overexpression of SMAD6 in *LMNA*^+/Q310*^ human myoblasts was responsible for a partial sequestration of phosphorylated R-SMADs in the cytoplasm (Fig. 7H). The quantification of grey levels of each labelling confirmed that, in myoblasts from the healthy control, phosphorylated R-SMADs (in green) were accumulated in the cells nuclei (DAPI staining in blue) whereas, in affected myoblasts, phosphorylated R-SMADs were diffuse in both the cytoplasm and nucleus of the cell, partially colocalizing with overexpressed SMAD6 dots (Fig. 7I). Finally, a premature expression of myogenin was found in proliferative *LMNA*^+/Q310*^ human myoblasts compared to those from the healthy control (Fig. 7G).

Altogether, these results demonstrate that the BMP4 pathway is impaired simultaneously in human myoblasts mutated for the *LMNA* gene and in murine *Lmna*^−/−^ myoblasts. In *Lmna*^−/−^ myoblasts, this impairment is responsible for an accelerated differentiation phenotype and Smad6 inactivation is sufficient to restore a normal response to recombinant Bmp4 and a timely differentiation.

## Discussion

Nuclear lamins play a crucial role in insuring the integrity of the nuclear envelope and the organization of chromatin regulating gene expression processes. *LMNA* mutations have been associated with several human pathologies, generally only affecting one or few tissues, suggesting that each particular mutation could alter the interaction between lamins A/C and tissue-specific partners^42^. Importantly, more than 80% of currently known mutations lead to skeletal muscle defects^43^. Lamins A/C have been shown to be necessary for proper muscle differentiation^20^. Primary *Lmna*-null myoblasts displayed impaired expression of muscle differentiation regulators such as MyoD1, pRB and Desmin and altered differentiation potential. However, it was suggested that the culture conditions required by primary myoblasts, based on FGF complementation, could interfere with the pathological phenotype and limit the identification of the altered signaling pathways in *Lmna*-null myoblasts^20^. To avoid the issues linked to primary cells culture and to tightly control the proliferation/differentiation transition, we used a previously described model of conditionally immortalized murine myoblasts, the H-2K cell lines, to identify and dissect new potentially altered signaling pathways in *LMNA*-mutated myoblasts^18^.

Our results reinforce the central role of muscle stem cells, so called satellite cells, in the muscular forms of laminopathies by showing *Lmna*^−/−^ H-2K myoblasts precocious differentiation due to the alteration of a crucial signaling pathway implicated in the maintenance of stem cells proliferation and the prevention of premature differentiation: the BMP4 pathway. They also identify Smad6 overexpression as the main defect of this pathway as siRNA-mediated Smad6 inactivation restored *Lmna*^−/−^ myoblasts response to Bmp4 and timely differentiation. Finally, these defects were also observed in human primary myoblasts from patients carrying heterozygous *LMNA* mutations, suggesting that BMP4 pathway alterations are relevant in human muscular laminopathies.

Our results on the consequence of BMP4 pathway alterations in *LMNA*-mutated myoblasts are consistent with recent data showing that BMP4 pathway is crucial for preventing premature myogenic differentiation^27^ and for the induction^44^ and the formation of myotubes^45^. BMP4 pathway is known to be important *in vivo* for muscle mass control: its inhibition is responsible for muscle atrophy and can abolish the hypertrophy observed in myostatin-deficient mice^28,46^. Moreover, BMPRII receptor has been shown to be enriched at the neuromuscular junction (NMJ) in drosophila^47^ and BMP pathway to be crucial for the differentiation of motor neurons and the successful assembly of NMJ^48^. Finally, BMP pathway is known to be important for heart embryogenesis and morphogenesis^49,50^. Interestingly, *LMNA* mutations have been associated, both in mice and humans, with muscle wasting^38^, dysfunctional neuromuscular junctions^9^ and dilated cardiomyopathy with conduction defects^51,52^. In this context, exploration and *in vivo* modulation of the BMP pathway could represent an interesting approach to rescue the muscle and cardiac phenotypes observed in *Lmna*^*∆*8–11^ mice and *LMNA*-mutated patients.

By competing with SMAD4 to interact with phosphorylated R-SMADs, SMAD6, has an inhibitory role on the BMP pathway and avoids the expression of target genes such as *ID1* and *ID2*^34^. SMADs are common intracellular transducers in at least two distinct activation pathways: the BMP4 pathway and the TGFβ pathway^18^. Alterations of the TGFβ pathway have been previously described in the same model of conditionally immortalized *Lmna*^−/−^ myoblasts. In this study, increased nuclear levels of Smad2/3 have been observed in *Lmna*^−/−^ H-2K MBs. This TGFβ pathway alteration was responsible for a delayed activation of the satellite cell. Interestingly, TGFβ pathway is relatively close to BMP4 pathway. SMAD2 and SMAD3, phosphorylated in response to the binding of a ligand to its receptor, are able to bind SMAD4 to translocate into the nucleus in order to activate target genes expression^32,53^. In a similar way, SMAD7, but not SMAD6, is able to compete with SMAD4 to avoid SMAD2/3 intranuclear translocation^54–56^. In our study, the overexpression of SMAD6 found both at the transcript and at the protein level is the determinant alteration of the BMP4 pathway. Moreover, a slight increase in Smad4 expression was observed in *Lmna*^−/−^ H-2K MBs (Fig. 3A and B)^18^. One could postulate that the competition between Smad6 and Smad4 to interact with pSmad1/5/8 would lead to an increased availability of Smad4 to interact with pSmad2/3. As a consequence, modulating Smad6 expression would indirectly affect the TGFβ pathway by increasing the intranuclear translocation of Smad4-pSmad2/3 complexes. The overexpression of SMAD6 would then be responsible for an impaired proliferation and a precocious differentiation due to an imbalance between BMP4 and TGFβ pathways. Recently, a similar balance between BMP and TGFβ pathways has been described as a key regulator of muscle mass^57^. Using AAV-mediated codelivery of activin and myostatin inhibitors, the authors induced a complete inhibition of pSMAD2/3 pathway and an activation of the pSMAD1/5/8 axis leading to a hypertrophy mediated by both an increased protein synthesis and a decreased ubiquitin-dependent protein degradation. It was consistent with the fact that, at the opposite, genetic disruption of Smad7 lead to skeletal muscle growth and regeneration impairment^58^.

The way lamins A/C could regulate Bmp4 and Smad6 expression is currently unclear. Of note, in *Lmna*^−/−^ myoblasts, the expression and the localization of other lamins such as lamin B1 is not affected (Supplementary Figures S10 and S11). Amongst the different hypotheses proposed to explain how ubiquitous lamin mutations could affect muscle tissue is the “structural hypothesis”. This hypothesis postulates that the loss of structural functions of lamins A/C is responsible for a higher cellular susceptibility to suffer from mechanical damages^58^. This hypothesis is supported by evidences on abnormal *LMNA*-mutated myoblasts alignment in a soft-gel matrix associated with an enhance activation of the yes-associated protein (YAP) signaling^17^. Data in human mesenchymal stem cells (MSC) and chondrogenesis show that YAP activity is responsible for a decrease in R-SMADs phosphorylation and expression of *ID1* and *ID2*^59^. Moreover, YAP signaling has been shown to enhance the nuclear localization of phosphorylated SMAD2/3, molecular transducers of the TGFβ pathway^60^. Altogether, these results suggest that the higher susceptibility to mechanical damages and impaired mechanotransduction could be linked to BMP4 pathway impairments.

In all cases, a recent study investigating the impact of different *LMNA* mutations on MSC differentiation capabilities found that BMP4 expression is often altered in these contexts. Interestingly, depending on the *LMNA* mutation, BMP4 expression could be either stimulated or repressed on a case-by-case basis^61^. We also had the opportunity to test if our key results could be reproduced in human myoblasts from patients carrying *LMNA* mutations. We confirmed that BMP4 and SMAD6 expression are dysregulated in mutated patient myoblasts. In *LMNA*^+/Q310*^ myoblasts, these impairments lead to a partial sequestration of phosphorylated R-SMADs in the cytoplasm. Unexpectedly, we found *BMP4* RNA expression to be increased in patient cells whereas the BMP4 protein level was reduced, suggesting a potential post-transcriptional regulation or an impaired protein stability in *LMNA*^+/Q310*^ myoblasts. This observation was in line with the absence of detection of Bmp4 protein in *Lmna*^−/−^ H-2K myoblasts.

The major specific role of BMP pathway in the maintenance of an adult satellite cell pool has been recently demonstrated. More particularly, the authors showed that the SMAD6 overexpression is capable of inhibiting satellite cell-dependent postnatal muscle growth and the generation of an adult satellite cell pool^62^. Based on these observations and our results, inactivation of SMAD6 expression appears as a potential therapeutic target to modulate the balance between BMP4 and TGFβ pathways. This could rescue satellite cells proliferation and differentiation, prevent satellite cells exhaustion and, more largely, improve the muscle phenotype observed in striated muscle laminopathies.

Taken together, these results provide a new insight into the pathophysiological mechanism underlying skeletal muscles and muscle stem cells defects in striated muscle laminopathies.

## Electronic supplementary material

Supplementary data

Supplementary Dataset 1

## References

[1] Aebi U, Cohn J, Buhle L, Gerace L (1986). The nuclear lamina is a meshwork of intermediate-type filaments. Nature.

[2] Lin F, Worman HJ (1993). Structural organization of the human gene encoding nuclear lamin A and nuclear lamin C. J. Biol. Chem..

[3] Burke B, Stewart CL (2006). The laminopathies: the functional architecture of the nucleus and its contribution to disease. Annu. Rev. Genomics Hum. Genet..

[4] Dechat T (2008). Nuclear lamins: major factors in the structural organization and function of the nucleus and chromatin. Genes Dev..

[5] Gruenbaum Y, Foisner R (2015). Lamins: nuclear intermediate filament proteins with fundamental functions in nuclear mechanics and genome regulation. Annu. Rev. Biochem..

[6] Janin A, Bauer D, Ratti F, Millat G, Méjat A (2017). Nuclear envelopathies: a complex LINC between nuclear envelope and pathology. Orphanet J. Rare Dis..

[7] Worman HJ (2012). Nuclear lamins and laminopathies. J. Pathol..

[8] Lattanzi G (2011). Laminopathies: many diseases, one gene. Report of the first Italian Meeting Course on Laminopathies. Acta Myol. Myopathies Cardiomyopathies Off. J. Mediterr. Soc. Myol. Ed. Gaetano Conte Acad. Study Striated Muscle Dis..

[9] Méjat A (2009). Lamin A/C-mediated neuromuscular junction defects in Emery-Dreifuss muscular dystrophy. J. Cell Biol..

[10] Sullivan T (1999). Loss of A-type lamin expression compromises nuclear envelope integrity leading to muscular dystrophy. J. Cell Biol..

[11] Mounkes LC, Kozlov SV, Rottman JN, Stewart CL (2005). Expression of an LMNA-N195K variant of A-type lamins results in cardiac conduction defects and death in mice. Hum. Mol. Genet..

[12] Wang Y, Herron AJ, Worman HJ (2006). Pathology and nuclear abnormalities in hearts of transgenic mice expressing M371K lamin A encoded by an LMNA mutation causing Emery-Dreifuss muscular dystrophy. Hum. Mol. Genet..

[13] Bertrand AT (2012). DelK32-lamin A/C has abnormal location and induces incomplete tissue maturation and severe metabolic defects leading to premature death. Hum. Mol. Genet..

[14] Kubben N (2011). Post-natal myogenic and adipogenic developmental: defects and metabolic impairment upon loss of A-type lamins. Nucl. Austin Tex.

[15] Zhang H, Kieckhaefer JE, Cao K (2013). Mouse models of laminopathies. Aging Cell.

[16] Broers JLV (2004). Decreased mechanical stiffness in LMNA^−/−^ cells is caused by defective nucleo-cytoskeletal integrity: implications for the development of laminopathies. Hum. Mol. Genet..

[17] Bertrand AT (2014). Cellular microenvironments reveal defective mechanosensing responses and elevated YAP signaling in LMNA-mutated muscle precursors. J. Cell Sci..

[18] Cohen TV (2013). Defective skeletal muscle growth in lamin A/C-deficient mice is rescued by loss of Lap2α. Hum. Mol. Genet..

[19] Bakay M (2006). Nuclear envelope dystrophies show a transcriptional fingerprint suggesting disruption of Rb-MyoD pathways in muscle regeneration. Brain J. Neurol..

[20] Frock RL (2006). Lamin A/C and emerin are critical for skeletal muscle satellite cell differentiation. Genes Dev..

[21] Jahn D (2012). A truncated lamin A in the Lmna^−/−^ mouse line: implications for the understanding of laminopathies. *Nucl. Austin*. Tex.

[22] Jat PS (1991). Direct derivation of conditionally immortal cell lines from an H-2Kb-tsA58 transgenic mouse. Proc. Natl. Acad. Sci. USA.

[23] Morgan JE (1994). Myogenic cell lines derived from transgenic mice carrying a thermolabile T antigen: a model system for the derivation of tissue-specific and mutation-specific cell lines. Dev. Biol..

[24] Chen G, Deng C, Li Y-P (2012). TGF-β and BMP Signaling in Osteoblast Differentiation and Bone Formation. Int. J. Biol. Sci..

[25] Wozney JM (1992). The bone morphogenetic protein family and osteogenesis. Mol. Reprod. Dev..

[26] Miyazono K, Kamiya Y, Morikawa M (2010). Bone morphogenetic protein receptors and signal transduction. J. Biochem. (Tokyo).

[27] Ono Y (2011). BMP signalling permits population expansion by preventing premature myogenic differentiation in muscle satellite cells. Cell Death Differ..

[28] Sartori R (2013). BMP signaling controls muscle mass. Nat. Genet..

[29] Muses S, Morgan JE, Wells DJ (2011). A new extensively characterised conditionally immortal muscle cell-line for investigating therapeutic strategies in muscular dystrophies. PloS One.

[30] Chen EY (2013). Enrichr: interactive and collaborative HTML5 gene list enrichment analysis tool. BMC Bioinformatics.

[31] Kuleshov MV (2016). Enrichr: a comprehensive gene set enrichment analysis web server 2016 update. Nucleic Acids Res..

[32] Feng X-H, Derynck R (2005). Specificity and versatility in tgf-beta signaling through Smads. Annu. Rev. Cell Dev. Biol..

[33] Imamura T (1997). Smad6 inhibits signalling by the TGF-beta superfamily. Nature.

[34] Hata A, Lagna G, Massagué J, Hemmati-Brivanlou A (1998). Smad6 inhibits BMP/Smad1 signaling by specifically competing with the Smad4 tumor suppressor. Genes Dev..

[35] Hollnagel A, Oehlmann V, Heymer J, Rüther U, Nordheim A (1999). Id genes are direct targets of bone morphogenetic protein induction in embryonic stem cells. J. Biol. Chem..

[36] Katagiri T (2002). Identification of a BMP-responsive element in Id1, the gene for inhibition of myogenesis. Genes Cells Devoted Mol. Cell. Mech..

[37] Jen Y, Weintraub H, Benezra R (1992). Overexpression of Id protein inhibits the muscle differentiation program: *in vivo* association of Id with E2A proteins. Genes Dev..

[38] Bonne G (2000). Clinical and molecular genetic spectrum of autosomal dominant Emery-Dreifuss muscular dystrophy due to mutations of the lamin A/C gene. Ann. Neurol..

[39] Quijano-Roy S (2008). De novo LMNA mutations cause a new form of congenital muscular dystrophy. Ann. Neurol..

[40] Muchir A (2004). Nuclear envelope alterations in fibroblasts from patients with muscular dystrophy, cardiomyopathy, and partial lipodystrophy carrying lamin A/C gene mutations. Muscle Nerve.

[41] Arimura T (2005). Mouse model carrying H222P-Lmna mutation develops muscular dystrophy and dilated cardiomyopathy similar to human striated muscle laminopathies. Hum. Mol. Genet..

[42] Scharner J, Gnocchi VF, Ellis JA, Zammit PS (2010). Genotype-phenotype correlations in laminopathies: how does fate translate?. Biochem. Soc. Trans..

[43] Bertrand AT, Chikhaoui K, Yaou RB, Bonne G (2011). Clinical and genetic heterogeneity in laminopathies. Biochem. Soc. Trans..

[44] Furutani Y, Umemoto T, Murakami M, Matsui T, Funaba M (2011). Role of endogenous TGF-β family in myogenic differentiation of C2C12 cells. J. Cell. Biochem..

[45] Umemoto T, Furutani Y, Murakami M, Matsui T, Funaba M (2011). Endogenous Bmp4 in myoblasts is required for myotube formation in C2C12 cells. Biochim. Biophys. Acta.

[46] Winbanks CE (2013). The bone morphogenetic protein axis is a positive regulator of skeletal muscle mass. J. Cell Biol..

[47] Chou H-J (2013). BMP4 is a peripherally-derived factor for motor neurons and attenuates glutamate-induced excitotoxicity *in vitro*. PloS One.

[48] Benavente F, Pinto C, Parada M, Henríquez JP, Osses N (2012). Bone morphogenetic protein 2 inhibits neurite outgrowth of motor neuron-like NSC-34 cells and up-regulates its type II receptor. J. Neurochem..

[49] Klaus A (2012). Wnt/β-catenin and Bmp signals control distinct sets of transcription factors in cardiac progenitor cells. Proc. Natl. Acad. Sci. USA.

[50] Kruithof BPT, Duim SN, Moerkamp AT, Goumans M-J (2012). TGFβ and BMP signaling in cardiac cushion formation: lessons from mice and chicken. Differ. Res. Biol. Divers..

[51] Millat G (2011). Clinical and mutational spectrum in a cohort of 105 unrelated patients with dilated cardiomyopathy. Eur. J. Med. Genet..

[52] Mounkes LC, Burke B, Stewart CL (2001). The A-type lamins: nuclear structural proteins as a focus for muscular dystrophy and cardiovascular diseases. Trends Cardiovasc. Med..

[53] Massagué J, Wotton D (2000). Transcriptional control by the TGF‐β/Smad signaling system. EMBO J..

[54] Huang Y (2008). [Smad7 instead of Smad6 blocks epithelial-mesenchymal transition induced by TGF-beta in human renal proximal tubule epithelial cells]. Xi Bao Yu Fen Zi Mian Yi Xue Za Zhi Chin. J. Cell. Mol. Immunol..

[55] Zhao J, Shi W, Chen H, Warburton D (2000). Smad7 and Smad6 Differentially Modulate Transforming Growth Factor β-induced Inhibition of Embryonic Lung Morphogenesis. J. Biol. Chem..

[56] Nakao A (1997). Identification of Smad7, a TGFbeta-inducible antagonist of TGF-beta signalling. Nature.

[57] Chen JL (2017). Specific targeting of TGF-β family ligands demonstrates distinct roles in the regulation of muscle mass in health and disease. Proc. Natl. Acad. Sci..

[58] Cohen TV, Kollias HD, Liu N, Ward CW, Wagner KR (2015). Genetic disruption of Smad7 impairs skeletal muscle growth and regeneration. J. Physiol..

[59] Karystinou A (2015). Yes-associated protein (YAP) is a negative regulator of chondrogenesis in mesenchymal stem cells. Arthritis Res. Ther..

[60] Nishio M (2016). Dysregulated YAP1/TAZ and TGF-β signaling mediate hepatocarcinogenesis in Mob1a/1b-deficient mice. Proc. Natl. Acad. Sci. USA.

[61] Malashicheva A (2015). Various lamin A/C mutations alter expression profile of mesenchymal stem cells in mutation specific manner. Mol. Genet. Metab..

[62] Stantzou, A. *et al*. BMP signaling regulates satellite cell dependent postnatal muscle growth. *Development* dev 144089, 10.1242/dev.144089 (2017). 10.1242/dev.144089 PMC5560039 28694257

