# Supplementary material for: SMAD6 overexpression leads to accelerated myogenic differentiation of LMNA mutated cells
Source: Sci Rep. 2018 Apr 4;8:5618. doi: 10.1038/s41598-018-23918-x (PMC5884786; doi:10.1038/s41598-018-23918-x)

SMAD6 overexpression leads to accelerated myogenic differentiation of *LMNA* mutated cells.

Alexandre Janin[1,2,3,4,9], Delphine Bauer[1,2,3], Francesca Ratti[1,2,3], Camille Valla[1,2,3], Anne Bertrand[5], Emilie Christin[1,2,3,], Emilie Chopin[6], Nathalie Streichenberger[1,2,3,7], Gisèle Bonne[5], Vincent Gache[1,2,3], Tatiana Cohen[8] and Alexandre Méjat[1,2,3,9]

1. Univ. Lyon, Université Claude Bernard Lyon 1, Institut NeuroMyoGène, F-69622, Villeurbanne, France
2. CNRS UMR 5310, F-69622 Villeurbanne, France
3. INSERM U1217, F-69622 Villeurbanne, France
4. Laboratoire de Cardiogénétique Moléculaire, Centre de Biologie et Pathologie Est, Hospices Civils de Lyon, F-69677 Bron, France
5. Sorbonne Université, INSERM UMRS_974, Center of Research in Myology, 75013 Paris, France
6. Centre de Biotechnologie Cellulaire, Centre de Biologie et Pathologie Est, Hospices Civils de Lyon, F-69677 Bron, France
7. Centre de Neuropathologie Est, Centre de Biologie et Pathologie Est, Hospices Civils de Lyon, F-69677 Bron, France
8. Research Center for Genetic Medicine, Children’s National Medical Center; 111 Michigan Avenue NW, Washington DC 20010, USA
9. Corresponding authors: alexandre.janin01@chu-lyon.fr and alexandre.mejat@ens-lyon.fr

Supplementary Dataset: Differentially expressed genes in *Lmna* vs WT myoblasts

Supplementary Table S1: Characteristics of the patients.

	**Gender**	***LMNA* mutation**	**Symptoms**	**Maximal CK level**	**Age of onset**
1	M	p.Lys32del	Abnormal gait, Diffuse muscle contraction	4X	14 months
2	F	p.Lys32del	Axial hypotonia, axial and proximal muscle weakness, motor delay	4X	11 months
3	F	p.Leu380Ser	Fetal immobility, hypotonia, talipes, axial and proximal muscle weakness	4X	Birth
4	M	p.His222Pro	Elbow flexion, axial and proximal muscle weakness, rigid spine, cardiac arrhythmia	Not tested	Birth
5	M	p.Gln310*	Cardiomyopathy, diffuse muscle pain, walking difficulties, chronic tiredness,	2X	38 years old

Supplementary Figure S2: Control of the population purity after extraction of human myoblasts with a desmin labelling (in green) by immunofluorescence assay.

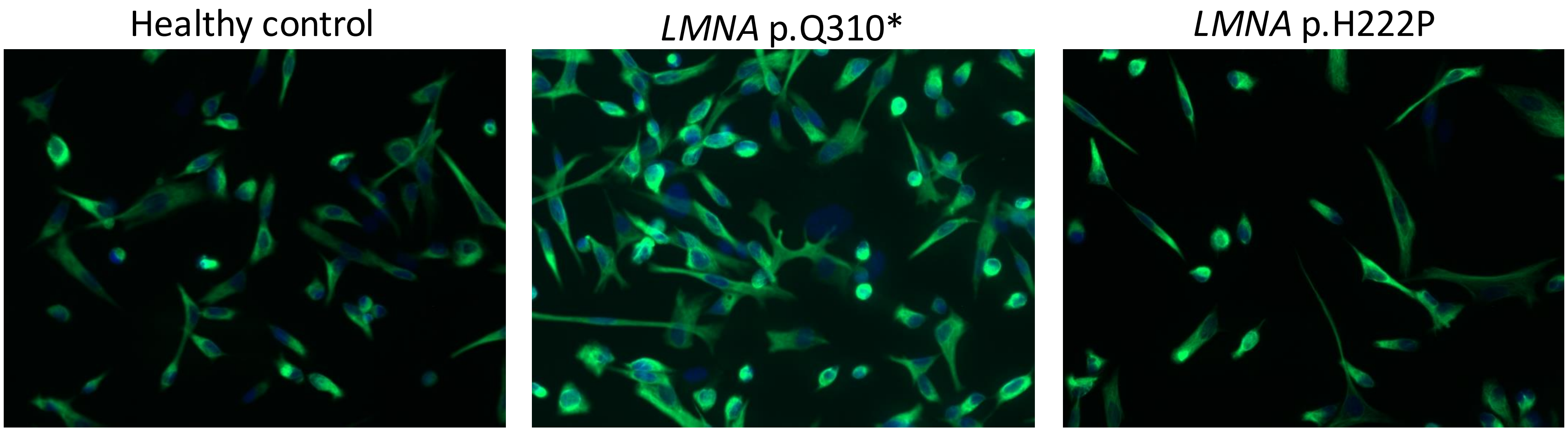

Supplementary Table S3: Sequences of primers used for RT-qPCR experiments.

Gene	Forward primer (5’-3’)	Reverse Primer (5’-3’)
**MOUSE**		
Bmp4	TTCCTGGACACCTCATCACA	CCACTCCCTTGAGGTAACGA
Bmp4 **(2nd couple)**	ACGTAGTCCCAAGCATCACC	ATGGCATGGTTGGTTGAGTT
Bmpr1a	ATGCAAGGATTCACCGAAAG	AGCAAAAGCAGCTGGAGAAG
Bmpr1b	TGACTCTGGAATGCCTGTTG	AGAGTGGGGTGGAGGTCTTT
Bmpr2	TGGCAGTGAGGTCACTCAAG	TTGCGTTCATTCTGCATAGC
Smad1	CAGCAGCTACCCCAACTCTC	CGTAAGCAACTGCCTGAACA
Smad4	CCCACTGAAGGACATTCGAT	GCCCTGAAGCTATCTGCAAC
Smad5	ATTGTTGGGCTGGAAACAAG	AAACTTGCAGACGTCCATCC
Smad6	ACGGTGACCTGCTGTCTCTT	AGCGAGTACGTGACCGTCTT
Smad8	CTTCACCGACCCTTCCAATA	TCTGGACAAAGATGCTGCTG
Id1	CATGAACGGCTGCTACTCAC	GTGGTCCCGACTTCAGACTC
Id2	CTCCAAGCTCAAGGAACTGG	ATGCTGATGTCCGTGTTCAG
Myog	CTACAGGCCTTGCTCAGCTC	ACGATGGACGTAAGGGAGTG
CycloB	GATGGCACAGGAGGAAAGAG	AACTTTGCCGAAAACCACAT
Hprt	AGGACCTCTCGAAGTGTTGG	TGGCAACATCAACAGGACTC
Ppib	AACTTTGGCATTGTGGAAGG	ACACATTGGGGGTAGGAACA
**HUMAN**		
LMNA	AGCAAAGTGCGTGAGGAGTT	TCAGGTCACCCTCCTTCTTG
BMP4	CCTAGCAAGAGTGCCGTCAT	GTTCTTCGTGGTGGAAGCTC
SMAD1	ACCTGCTTACCTGCCTCCTG	CATAAGCAACCGCCTGAACA
SMAD4	CATCCTGCTCCTGAGTATTGG	GGGTCCACGTATCCATCAAC
SMAD6	TACCACTTCAGCCGGCTCTG	AGTACGCCACGCTGCACCAG
ID1	GCTGCTCTACGACATGAACG	CCAACTGAAGGTCCCTGATG
HPRT	TGCTCGAGATGTGATGAAGG	TCCCCTGTTGACTGGTCATT
GAPDH	GAGTCAACGGATTTGGTCGT	TTGATTTTGGAGGGATCTCG

Supplementary Table S4: List of used antibodies.

Protein	Supplier	Reference
**BMP4**	Abcam	Ab39973
**BMPR1A**	Abcam	Ab38560
**BMPR1B**	Abcam	Ab175385
**BMPR2**	Cell Signaling	#6979
**ID1**	Abcam	Ab134163
**LAMIN A/C**	SantaCruz	Sc6215
**LAMIN B**	SantaCruz	Sc6217
**pSMAD1/5/9**	Cell Signaling	#13820
**SMAD1**	Cell Signaling	#6944
**SMAD4**	Cell Signaling	#9515
**SMAD6**	Abcam	Ab13727
**MYOGENIN**	Dako	M3559

Supplementary Table S5: Enriched GO biological processes in downregulated gene set

**TOP 24 GO Biological Processes enriched in the downregulated gene set**							
Term	Overlap	P-value	Adjusted P-value	Old P-value	Old Adjusted P-value	Z-score	Combined Score
muscle filament sliding (GO:0030049)	15/38	4,60551E-23	2,06788E-20	7,23769E-21	3,24972E-18	-2,485464424	112,654123
skeletal muscle contraction (GO:0003009)	8/17	1,36583E-13	3,0663E-11	4,06828E-12	9,13329E-10	-2,791631654	67,57972348
cardiac muscle contraction (GO:0060048)	7/33	3,16408E-09	4,73558E-07	9,90408E-09	1,48231E-06	-2,812158767	40,95344606
muscle contraction (GO:0006936)	8/85	1,79108E-07	2,01049E-05	2,59975E-07	2,91822E-05	-2,599239354	28,10959666
regulation of ATPase activity (GO:0043462)	3/8	1,99363E-05	0,001491896	5,19866E-05	0,003870666	-2,556517204	16,63706603
sarcomere organization (GO:0045214)	4/28	4,61347E-05	0,002589307	6,86158E-05	0,003870666	-2,296022284	13,67594653
cardiac myofibril assembly (GO:0055003)	3/9	2,97465E-05	0,001908024	6,89651E-05	0,003870666	-1,922844334	12,04024927
skeletal muscle tissue development (GO:0007519)	3/19	0,000325484	0,01623802	0,000458939	0,022895945	-2,306655923	9,504344777
striated muscle contraction (GO:0006941)	3/21	0,000442054	0,019848247	0,000597115	0,026810444	-2,38487726	9,347859342
type I interferon signaling pathway (GO:0060337)	4/62	0,001040045	0,031604026	0,001147665	0,039638594	-2,683642894	9,270565888
positive regulation of B cell proliferation (GO:0030890)	3/23	0,000582458	0,021793627	0,00075934	0,030994876	-2,251270142	8,613669539
positive regulation of apoptotic process (GO:0043065)	6/231	0,006617016	0,076180519	0,00623454	0,075656989	-3,156974903	8,128103872
positive regulation of ERK1 and ERK2 cascade (GO:0070374)	5/148	0,004380498	0,056784163	0,004304687	0,05856983	-2,776825578	7,965318107
positive regulation of bone mineralization (GO:0030501)	3/30	0,001286979	0,033110354	0,0015382	0,046043448	-2,280533916	7,771852567
nervous system development (GO:0007399)	6/247	0,009052455	0,092442673	0,008480798	0,090663764	-3,032366047	7,220568678
heart development (GO:0007507)	4/79	0,002549436	0,043511702	0,002674305	0,052207086	-2,25694708	7,074909272
positive regulation of transcription, DNA-templated (GO:0045893)	8/464	0,019284993	0,141950196	0,017143845	0,1374569	-3,558112099	6,946427579
positive regulation of epithelial cell proliferation (GO:0050679)	3/32	0,00155583	0,034928393	0,001826735	0,046824017	-1,934657821	6,489723035
regulation of striated muscle contraction (GO:0006942)	2/8	0,001401106	0,033110354	0,002085702	0,046824017	-1,56884597	5,346484653
cellular response to interleukin-6 (GO:0071354)	2/9	0,001792923	0,036591937	0,002537639	0,051790907	-1,576319438	5,214350214
positive regulation of protein phosphorylation (GO:0001934)	4/113	0,00905897	0,092442673	0,009019704	0,094182491	-2,110522757	5,025506247
regulation of the force of heart contraction (GO:0002026)	2/11	0,002713425	0,043511702	0,003566314	0,057188398	-1,561015131	4,893353735
platelet degranulation (GO:0002576)	4/122	0,011762877	0,105806521	0,011615805	0,110021413	-1,962711065	4,408529974
apoptotic signaling pathway (GO:0097190)	3/60	0,009272053	0,092514481	0,009632532	0,096111263	-1,722794121	4,100922067

Supplementary Table S6: Enriched GO biological processes in upregulated gene set

Term	Overlap	P-value	Adjusted P-value	Old P-value	Old Adjusted P-value	Z-score	Combined Score
extracellular matrix organization (GO:0030198)	9/142	8,07029E-06	0,004559713	1,47853E-05	0,00835372	-2,983793958	16,08412823
negative regulation of apoptotic process (GO:0043066)	9/323	0,00358124	0,165340618	0,004739409	0,236465732	-4,584891087	8,251646654
positive regulation of cell proliferation (GO:0008284)	9/326	0,003804297	0,165340618	0,005022281	0,236465732	-4,51742088	8,130217311
positive regulation of cell migration (GO:0030335)	7/144	0,000431361	0,097485905	0,000630174	0,178024128	-3,118805915	7,260728246
negative regulation of calcium ion transport (GO:0051926)	2/8	0,002371056	0,148849616	0,003915212	0,236465732	-3,374987589	6,428739718
positive regulation of angiogenesis (GO:0045766)	5/77	0,00079523	0,097485905	0,001143384	0,210400217	-2,684285766	6,249144708
positive regulation of gene expression (GO:0010628)	6/214	0,01589623	0,237381949	0,019433041	0,265201106	-3,052492172	4,389742703
mitochondrial translational termination (GO:0070126)	4/87	0,009182494	0,212659302	0,011415406	0,265201106	-2,832242632	4,384492623
embryonic skeletal system development (GO:0048706)	2/10	0,003763743	0,165340618	0,005670567	0,246451567	-2,323704348	4,182081285
mitochondrial translational elongation (GO:0070125)	4/87	0,009182494	0,212659302	0,011415406	0,265201106	-2,675208111	4,141393148
negative regulation of canonical Wnt signaling pathway (GO:0090090)	5/140	0,010463235	0,21895287	0,012949129	0,265201106	-2,707187479	4,111943748
regulation of apoptotic process (GO:0042981)	5/155	0,015679627	0,237381949	0,019050487	0,265201106	-2,852879126	4,102682208
negative regulation of cell migration (GO:0030336)	4/78	0,006279581	0,197109072	0,00797859	0,253358786	-2,472639889	4,015562335
execution phase of apoptosis (GO:0097194)	3/37	0,005021537	0,189144545	0,006598143	0,250846102	-2,344257394	3,903760022
positive regulation of macrophage activation (GO:0043032)	2/8	0,002371056	0,148849616	0,003915212	0,236465732	-2,019923755	3,847588689
positive regulation of apoptotic signaling pathway (GO:2001235)	2/9	0,003029682	0,165340618	0,00475526	0,236465732	-2,136995608	3,846052682
cellular response to UV (GO:0034644)	3/40	0,006255198	0,197109072	0,008071607	0,253358786	-2,277413016	3,698514277
negative regulation of cysteine-type endopeptidase activity involved in apoptotic process (GO:0043154	3/45	0,00868177	0,212659302	0,010926756	0,265201106	-2,156784302	3,338839956
positive regulation of microtubule polymerization (GO:0031116)	2/15	0,008515137	0,212659302	0,011324193	0,265201106	-2,12361531	3,287492236
proteolysis (GO:0006508)	5/175	0,02499537	0,238386173	0,029766942	0,265201106	-2,237109848	3,207709811
negative regulation of cell proliferation (GO:0008285)	6/276	0,046583065	0,238386173	0,054572498	0,265201106	-2,200130482	3,154686452
positive regulation of epidermal growth factor-activated receptor activity (GO:0045741)	2/8	0,002371056	0,148849616	0,003915212	0,236465732	-1,548106903	2,948863092
cellular response to mechanical stimulus (GO:0071260)	3/47	0,009786092	0,212659302	0,012211971	0,265201106	-1,859336853	2,878372292
negative regulation of ossification (GO:0030279)	2/11	0,004571777	0,18450386	0,006659631	0,250846102	-1,622035674	2,741377991

Supplementary Figure S7: *Lmna* knock-down in H-2K wild-type myoblasts recapitulates decrease in Bmp4 expression and Smad6 overexpression found in *Lmna*$^{-/-}$ H-2K cells. Expression is shown as mean (± SEM) of biological replicates (n=3). Values are relative expression normalized on *Hprt1* housekeeping gene expression.

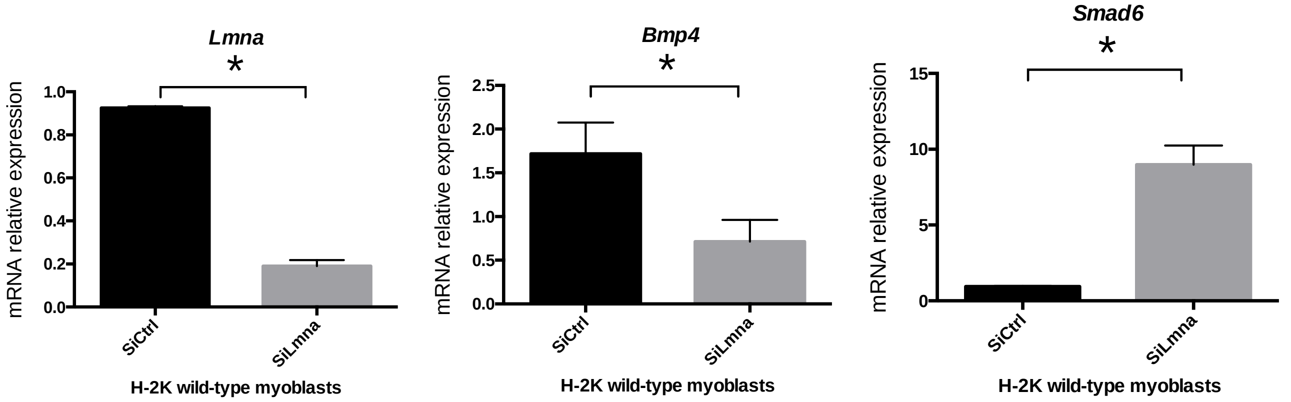

Supplementary Figure S8: *Lmna* overexpression in H-2K *Lmna*$^{-/-}$ myoblasts is able to reach *Bmp4* and *Smad6* expression found in wild-type H-2K cells, at the proliferation stage. Expression is shown as mean (± SEM) of biological replicates (n=3). Values are relative expression normalized on *Hprt1* housekeeping gene expression. Statistics are comparison to H-2K WT expression level (**p*<0.05, ***p*<0.01).
(Cells were seeded at 100.000 cells per wells of 6-well plated, transfected thanks to JetPrime™ reagent twenty-four after according to manufacturer's instructions and collected twenty-four hours later. NT : non transfected cells, 500ng and 1000ng : quantity of plasmid encoded *LMNA* cDNA used for transfection.)

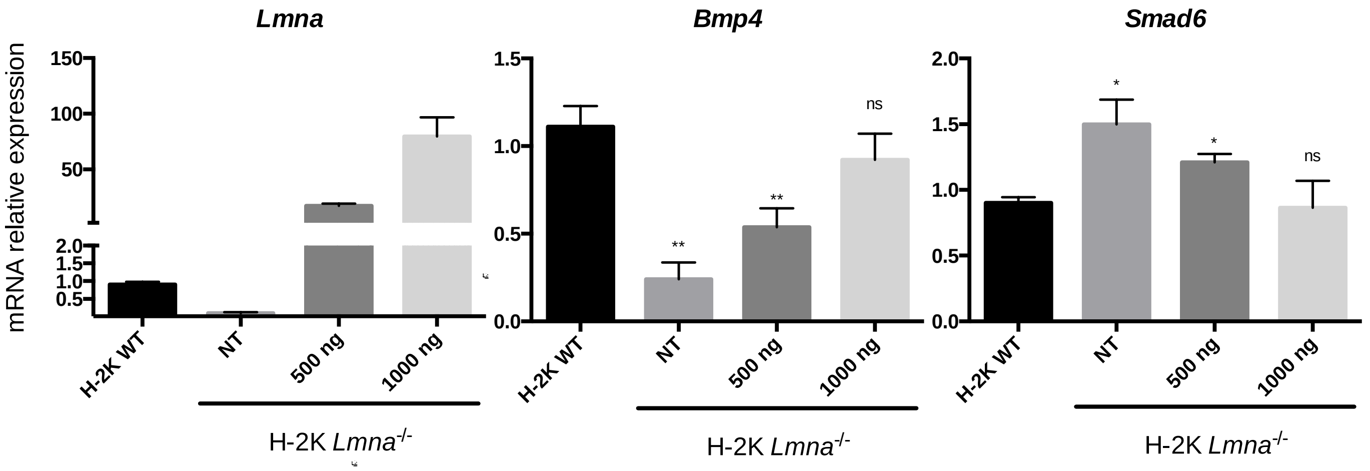

Supplementary Figure S9: Expression of *BMP4* and *SMAD6* measured by quantitative reverse transcription polymerase chain reaction (qRT-PCR) in MB from a healthy control and from carriers of various *LMNA* mutations (p.H222P, p.ΔK32 from 2 different patients and p.L380S). Expression is shown as mean (± SEM) of biological replicates (n=3). Values are relative expression normalized on *HPRT* housekepping gene expression. Statistics are comparison to healthy control expression level (*p<0.05, **p<0.01, ***p<0.001, **** p<0.0001).

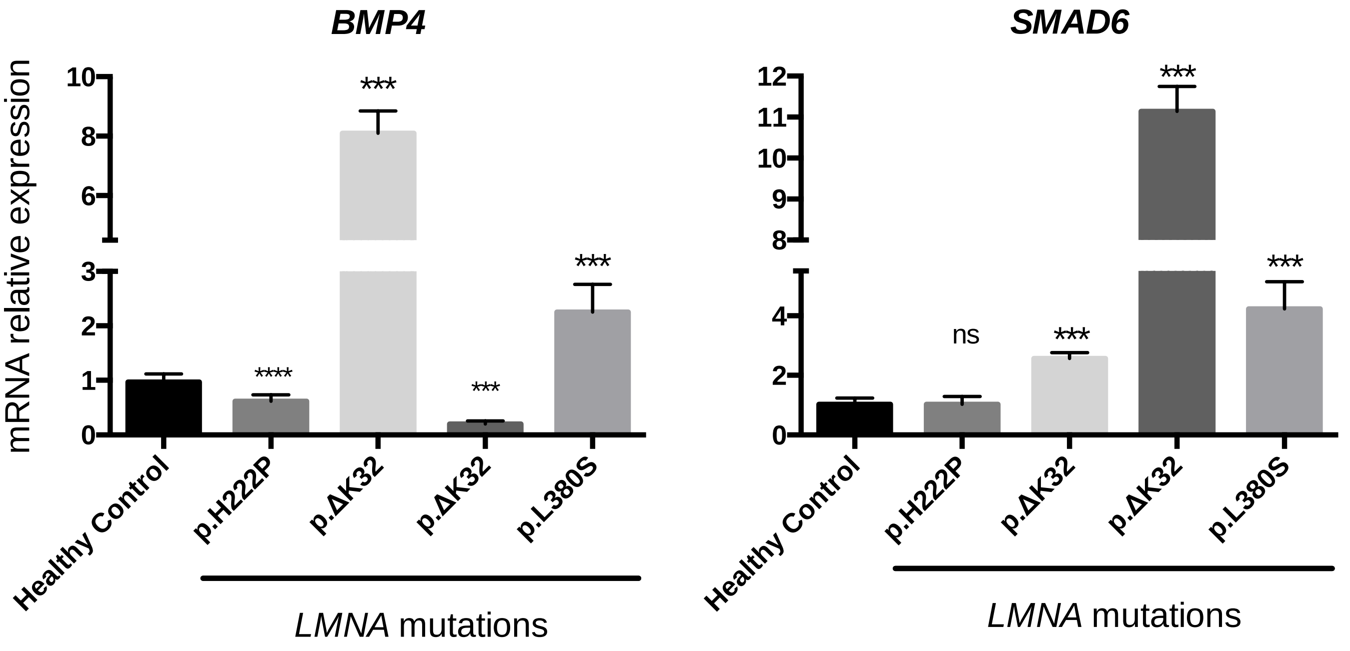

Supplementary Figure S10: Lamin B1 expression of *Lmna*$^{-/-}$ H-2K myoblasts.

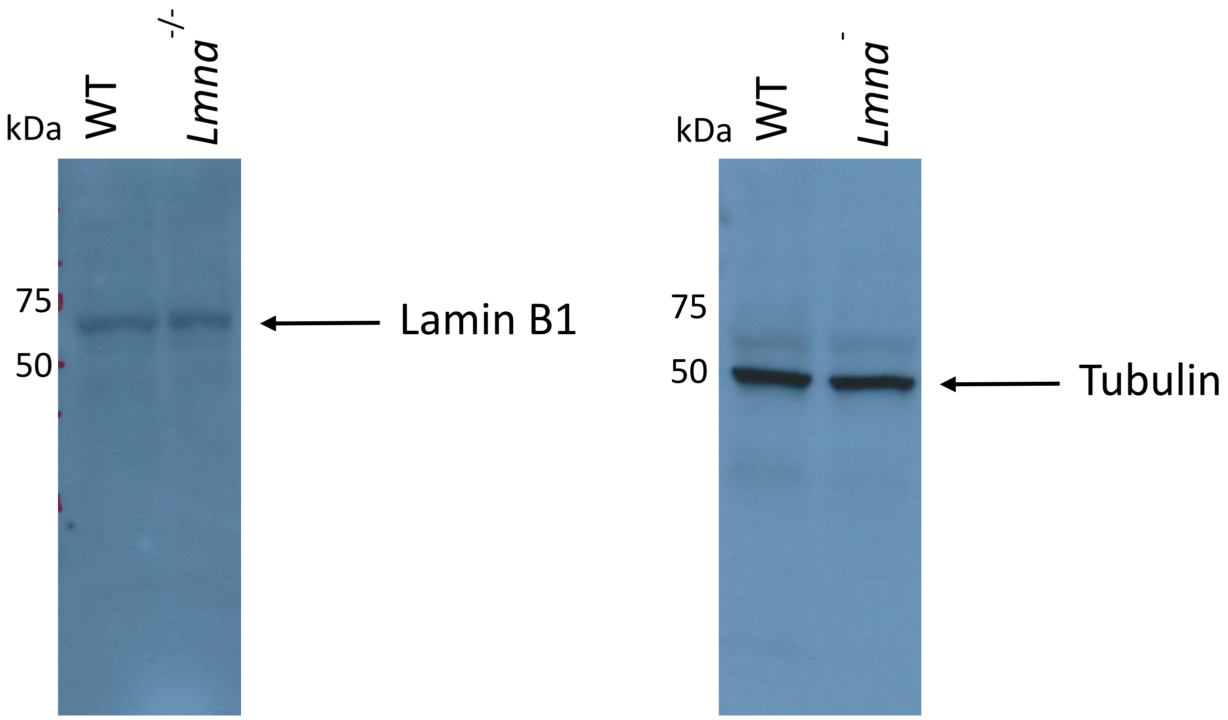

Supplementary Figure S11: Lamins A/C and B1 localisation in wild-type and *Lmna*$^{-/-}$ H-2K myoblasts. Lamin A/C (left) or lamin B1 (right) was labelled in red, nuclei were stained by DAPI in blue.

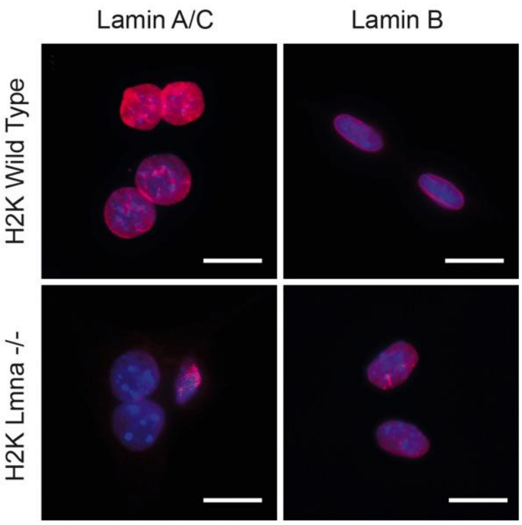

Unprocessed original scans of western blots shown in figure 2-7

Figure 2. Bmp4 and Bmp receptors (BmpR) expression in *Lmna*$^{-/-}$ vs wild-type H-2K myoblasts.

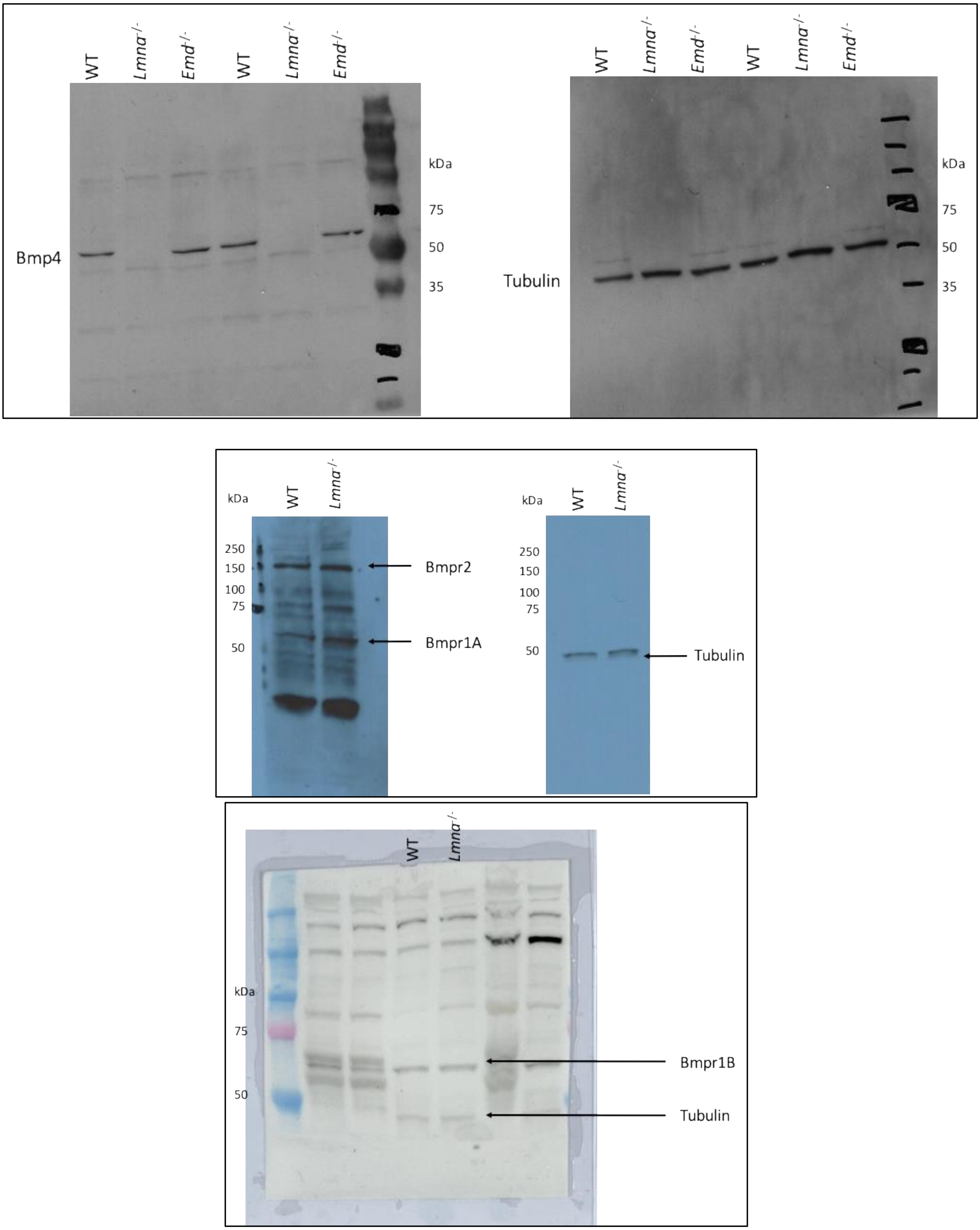

Figure 3. Smads expression and intracellular localization.

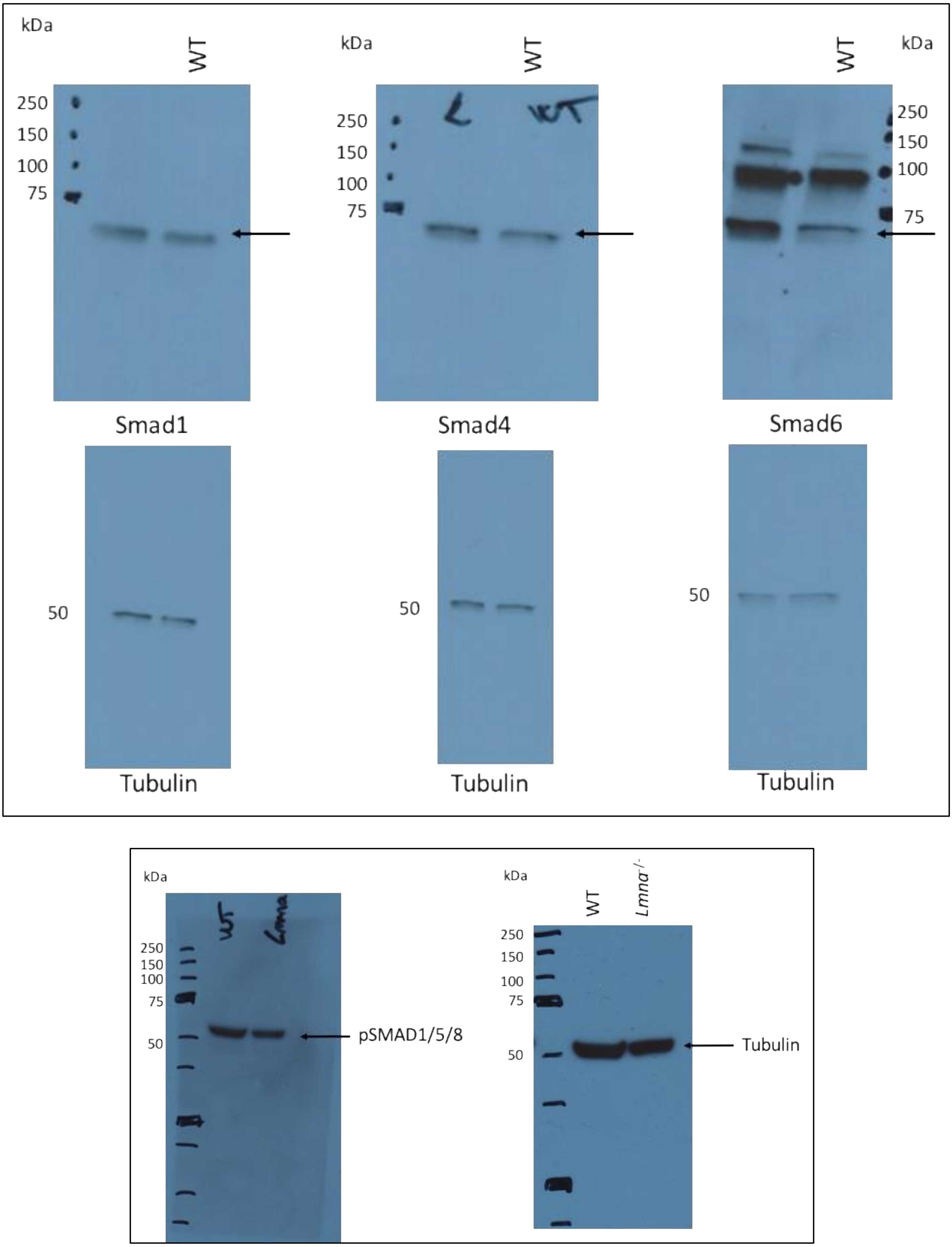

Figure 4. *Lmna*$^{-/-}$ myoblasts premature differentiation.

H-2K wild-type samples. Six time-points : proliferation and after 6, 12, 18, 24 and 48 hours of differentiation.

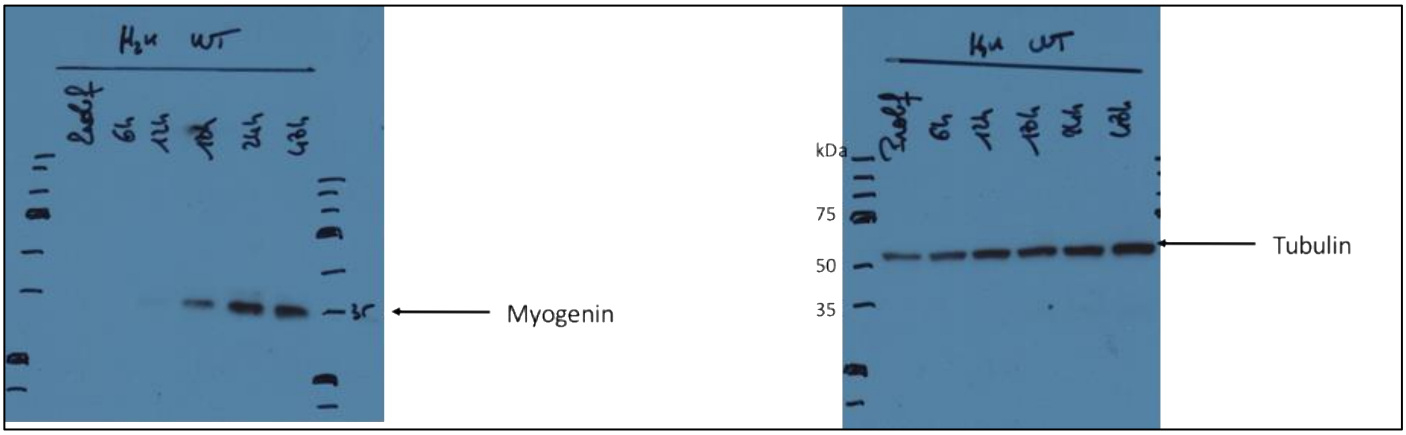

H-2K *Lmna*$^{-/-}$ samples. Six time-points : proliferation and after 6, 12, 18, 24 and 48 hours of differentiation.

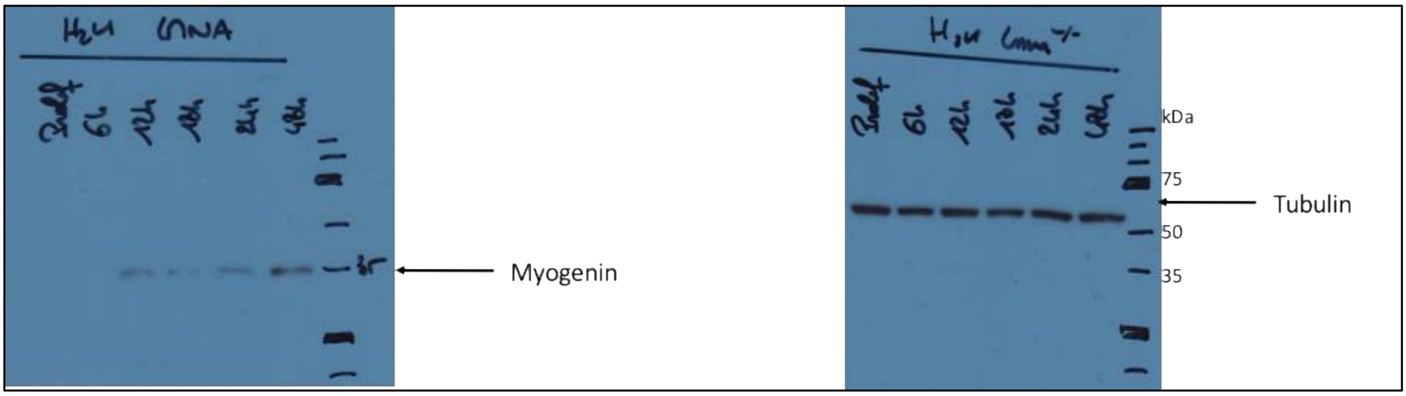

Figure 5. Wild-type and *Lmna*$^{-/-}$ myoblasts response to recombinant Bmp4 addition.

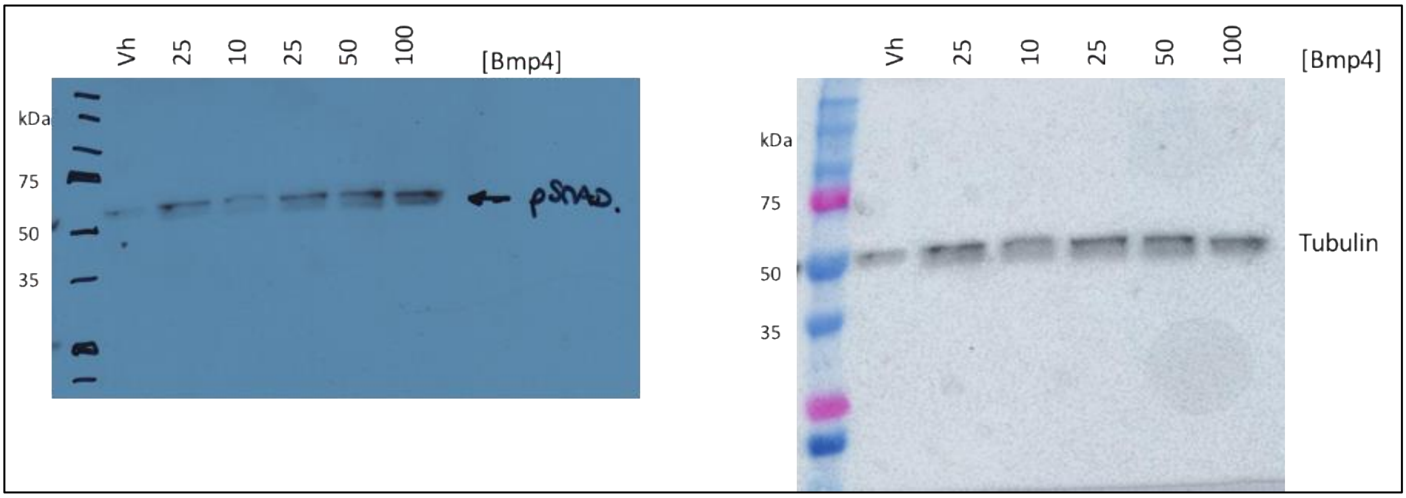

pSMAD 1/5/8 Tubulin

Figure 6. Precocious differentiation rescue in *Lmna*$^{-/-}$ MB by *Smad6* inactivation.

4 Samples : 1/ H-2K Wild-type (WT) + Control siRNA

2/ H-2K Wild-type (WT) + siRNA against Smad6 (Si1)

3/ H-2K *Lmna*$^{-/-}$ + Control siRNA

4/ H-2K *Lmna*$^{-/-}$ + siRNA against Smad6 (Si1)

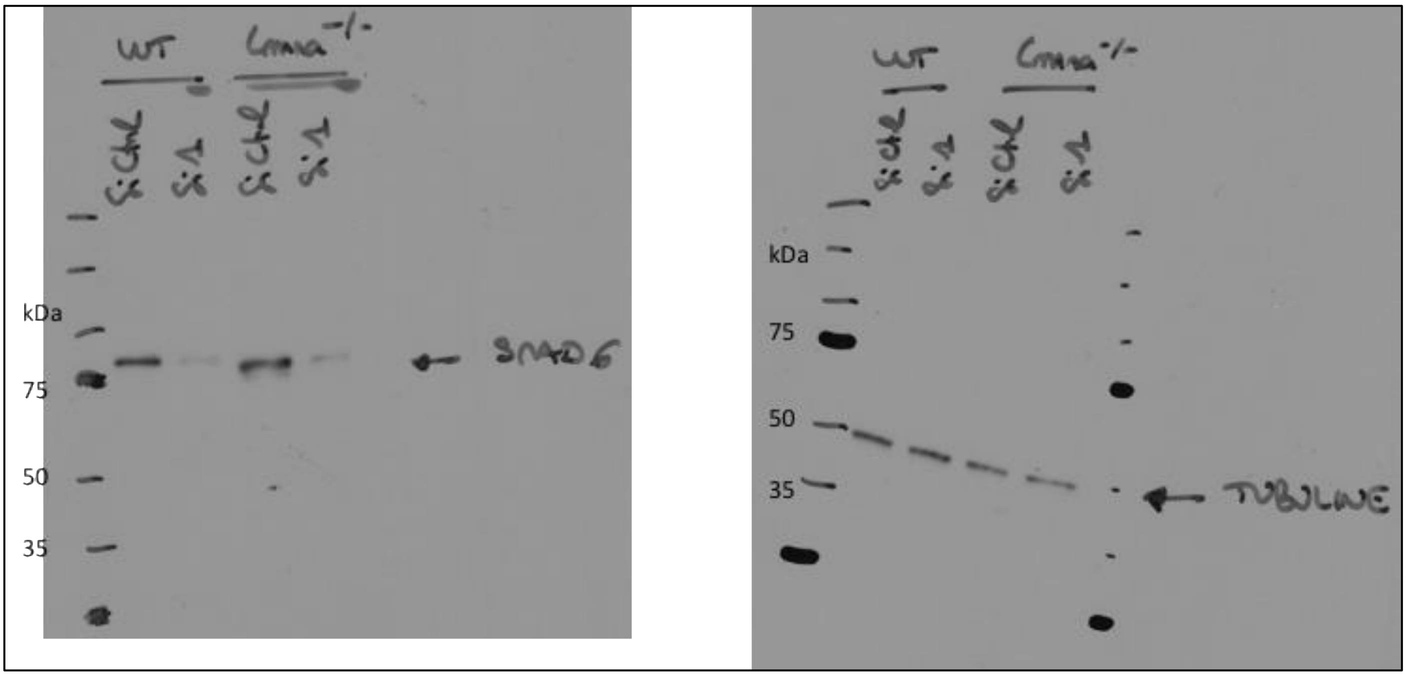

Smad6 Tubulin

Figure 7. Impaired BMP4 pathway in Human myoblasts carrying a false-sense mutation (*LMNA* p.Gln310*)

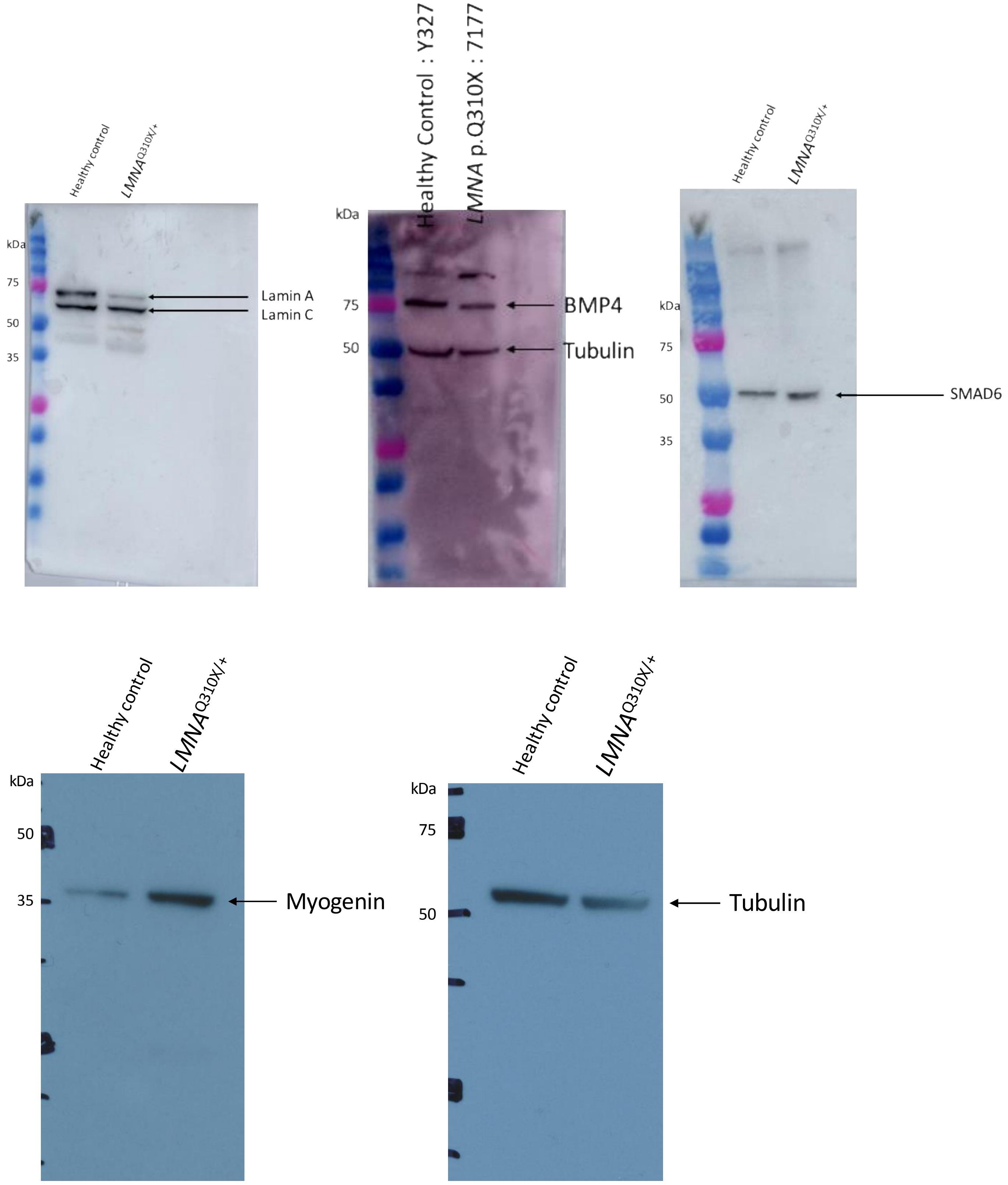

Supplement: Supplementary file 1 — Supplementary data [file 41598_2018_23918_MOESM1_ESM.pdf]
